# Recent advances in the structures and bioactivities of benzopyrans derived from marine fungi: a review

**DOI:** 10.3389/fphar.2024.1482316

**Published:** 2024-10-24

**Authors:** Yidan Xi, Huannan Wang, Lixiang Sun, Xueyang Ma, Shuncun Zhang, Zhen Zhang

**Affiliations:** ^1^ School of Pharmaceutical Sciences and Institute of Materia Medica, Shandong First Medical University and Shandong Academy of Medical Sciences, Jinan, China; ^2^ School of Pharmacy, Jining Medical University, Rizhao, China

**Keywords:** marine fungi, secondary metabolites, isolation and identification, benzopyrans, biological activity

## Abstract

Marine fungi represent a treasure trove of bioactive secondary metabolites, with benzopyran compounds emerging as a significant class of these natural products. This review delves into the structural diversity, biological activities, and sources of benzopyran compounds, highlighting their isolation from marine fungi inhabiting diverse environments such as sponges, marine sediments, algae, mangroves, and corals. Our literature search, conducted from 2000 to 2023, has identified a wealth of benzopyran compounds, showcasing their potential as lead compounds in drug development. The characteristics of benzopyran from marine fungi are explored, encompassing various subclasses such as chromones, isocoumarins, citrinins, and other related compounds. These compounds exhibit a remarkable chemical diversity, which is crucial for their diverse biological activities. The potential of benzopyran compounds in drug development is also discussed, emphasizing their roles in anti-tumor, antibacterial, anti-inflammatory, and enzyme inhibitory activities. In recent years, a remarkable 210 bioactive benzopyran compounds have been isolated from the secondary metabolites of marine fungi. These findings underscore the importance of marine fungi as a source of novel bioactive compounds, offering a plethora of potential lead compounds for the development of marine-derived drugs. This review aims to provide a comprehensive overview of the current state of research on benzopyran compounds, setting the stage for future advancements in the field of marine natural products.

## 1 Introduction

The ocean, a cornerstone of our planet’s ecological system, is a cradle of innumerable life forms and a bountiful reservoir of biological diversity and bioactive compounds. In this vast aquatic expanse, marine life has developed distinctive survival tactics and metabolic pathways, bequeathing humanity a wealth of pharmaceutical resources and potential lead compounds for drug discovery. In particular, the evolution of marine biotechnology and extensive research over recent decades have propelled marine-derived secondary metabolites to the forefront as a significant source for the innovation of novel pharmaceuticals (Blunt et al., 2017; Haque et al., 2022).

Benzopyran compounds, integral to a multitude of bioactive natural products, are composed of a benzene ring annelated to an oxygenated six-membered heterocyclic moiety. Celebrated for their structural complexity and pronounced bioactivity, these secondary metabolites have been unearthed in marine life, with a notable prevalence in marine fungi, thereby igniting new avenues of research in medicinal chemistry. These marine fungi, as microorganisms thriving in the marine milieu, have carved out distinctive ecological niches and adaptive strategies, culminating in the biosynthesis of an array of innovative and pharmacologically diverse secondary metabolites. The benzopyran class, in particular, has captured the spotlight in drug development, attributable to their broad spectrum of pharmacological actions, such as antibacterial, antifungal, antiviral, antitumor, antioxidant, and anti-inflammatory capabilities (Kumla et al., 2018; Pang et al., 2018a; Frank et al., 2019; Yang et al., 2020b; Wu et al., 2021).

In recent years, the exploration into the secondary metabolites of marine fungi has yielded a growing catalog of benzopyran compounds, with their biological activities being the subject of systematic investigation. Yet, despite the surge in research focusing on these marine fungal-derived benzopyran compounds, there remains a dearth of studies delving into their intricate chemical structures, biosynthetic pathways, and the mechanisms behind their bioactivity. This review endeavors to offer an exhaustive overview of the benzopyran compounds isolated from marine fungi over the past 20 years, encompassing their chemical structures, origins, biological activities, and the promise they hold for drug development. By meticulously examining and synthesizing the existing body of literature, this article aims to present a comprehensive vista of the research on benzopyran compounds from marine fungi to the reader, thereby catalyzing further advancements in scientific inquiry and tangible applications within this burgeoning domain.

## 2 Literature search

### 2.1 Methods

The literature search was conducted using previously reported methods (Soosaraei et al., 2018; Sun et al., 2019), where the selection of original articles is crucial because these papers have a direct impact on the research outcomes and the final conclusions. This review encompasses articles related to the target topic from the years 2000–2023, retrieved from the Web of Science and PubMed databases. The following descriptors were used during the search: benzopyran; chemical structure; biological activity.

### 2.2 Quantification of the studies

**FIGURE 1:**
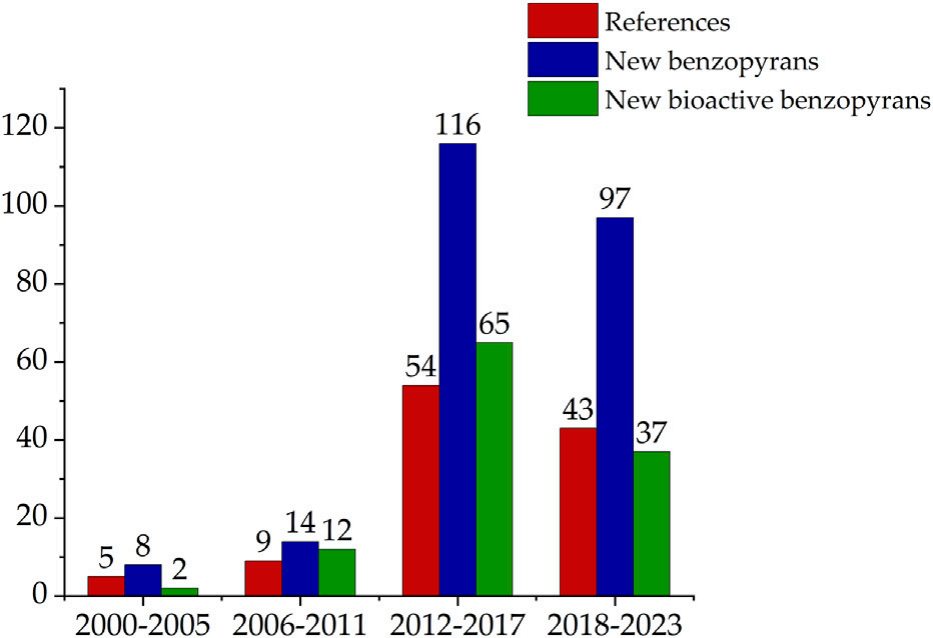
The annual isolation of novel and bioactive benzopyran compounds from marine fungi.

After conducting a literature search focused on the field of natural product chemistry, a total of 112 original articles indexed in the Web of Science and PubMed databases over the past 2 decades were identified. These studies have led to the isolation and identification of 510 benzopyran compounds from marine-derived fungi, with 289 novel compounds being characterized. Among these newly identified compounds, 116 have demonstrated biological activity. The number of references and compounds was further collated according to the year of publication, as illustrated in Figure 1. The search results indicate a significant upswing in the number of benzopyran compounds isolated and characterized in the last decade (2012-2023).

## 3 Characteristics of benzopyran from marine fungi

**FIGURE 2:**
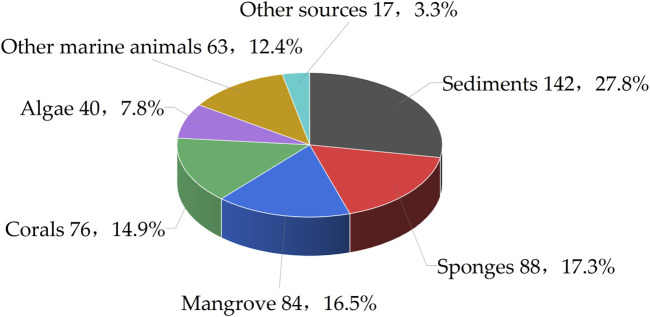
Principal origins of marine fungi yielding benzopyrans.

Marine fungi have yielded a rich collection of natural substances, playing a pivotal role in the discovery of bioactive secondary metabolites for drug development. Among these metabolites, benzopyran compounds constitute an important class, renowned for their diverse biological activities and potential therapeutic applications. Herein, this review encompasses an analysis of 510 benzopyran compounds isolated from a total of 112 distinct strains of marine fungi. Furthermore, the research reveals that a substantial portion of these benzopyran compounds, approximately 44.6%, originate from marine animals, with sponges accounting for 17.3% and corals for 14.9%. Additionally, marine plants such as algae and mangroves contribute to 24.3% of the benzopyran compounds, with 7.8% and 16.5% respectively. The remaining benzopyran compounds were isolated from various other components of the marine ecosystem, with 27.8% derived from marine sediments and 3.3% from other unspecified sources (Figure 2).

**FIGURE 3:**
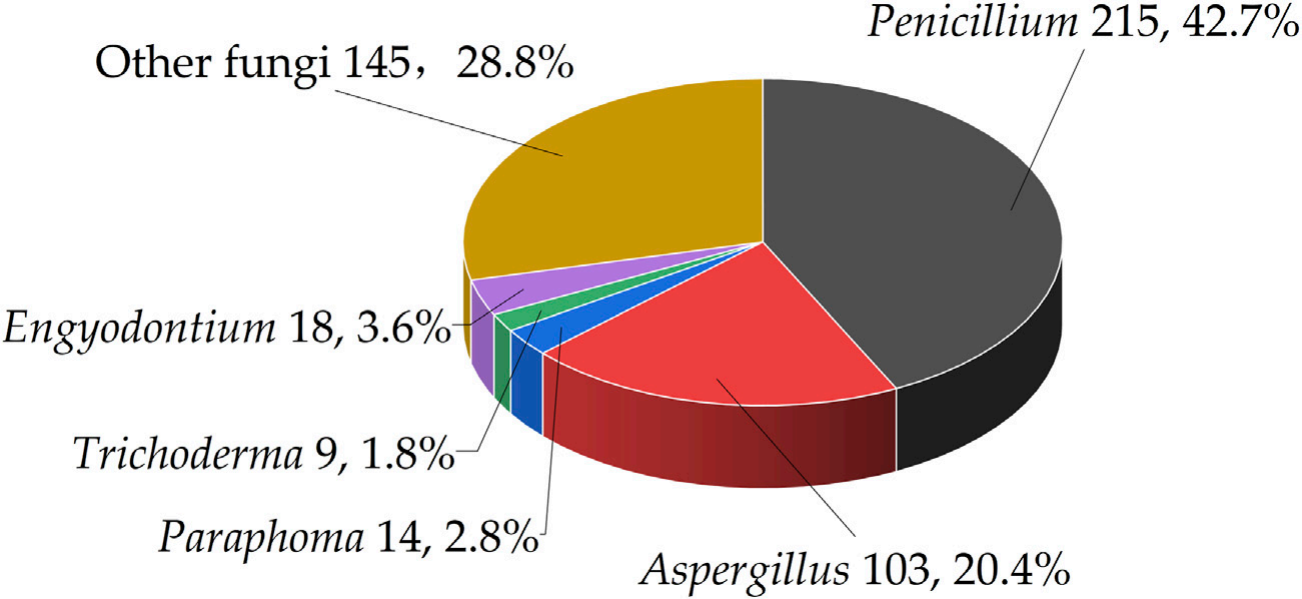
The distribution of marine fungi analyzed in this study.

This article presents a taxonomic classification at the genus level for 112 marine fungi to assess their distribution in the biosynthesis of benzopyran compounds. The results indicate that *Penicillium* is predominant, accounting for 42.7% of the evaluated species. It is followed by *Aspergillus*, which constitutes 20.4% of the total. In addition, *Paraphoma*, *Engyodontium*, and *Trichoderma* are represented by 2.8%, 3.6%, and 1.8% respectively. The remaining 28.8% are categorized under other fungal genera (Figure 3).

**FIGURE 4:**
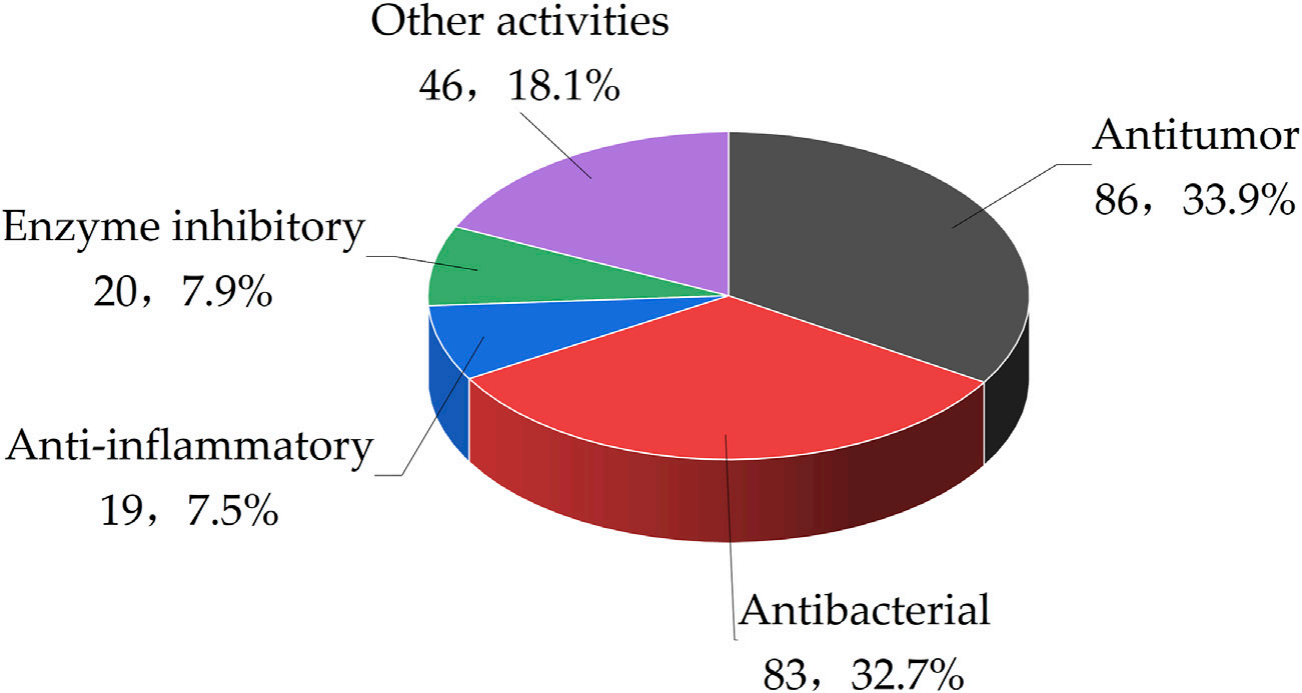
The bioactivity of Benzopyrans reviewed in this paper.

Recent research has highlighted the divergent metabolic processes in marine fungi, which result in the generation of a diverse array of secondary metabolites with unique chemical compositions and targeted biological effects. This article concludes that out of 510 benzopyran compounds isolated from marine fungi, 210 exhibit significant bioactivity. These benzopyran compounds have demonstrated a range of biological effects, including antitumor, antibacterial, anti-inflammatory, enzyme inhibitory, and antiviral properties. Specifically, 33.9% of the benzopyran compounds have shown antitumor activity, followed by antibacterial activity at 32.7%. Anti-inflammatory activities were observed in 7.5% of the compounds, while 7.9% displayed enzyme inhibitory activities. The remaining 18.1% of benzopyran compounds are associated with other bioactivities (Figure 4).

## 4 Chemical diversity

### 4.1 Chromones

**FIGURE 5:**
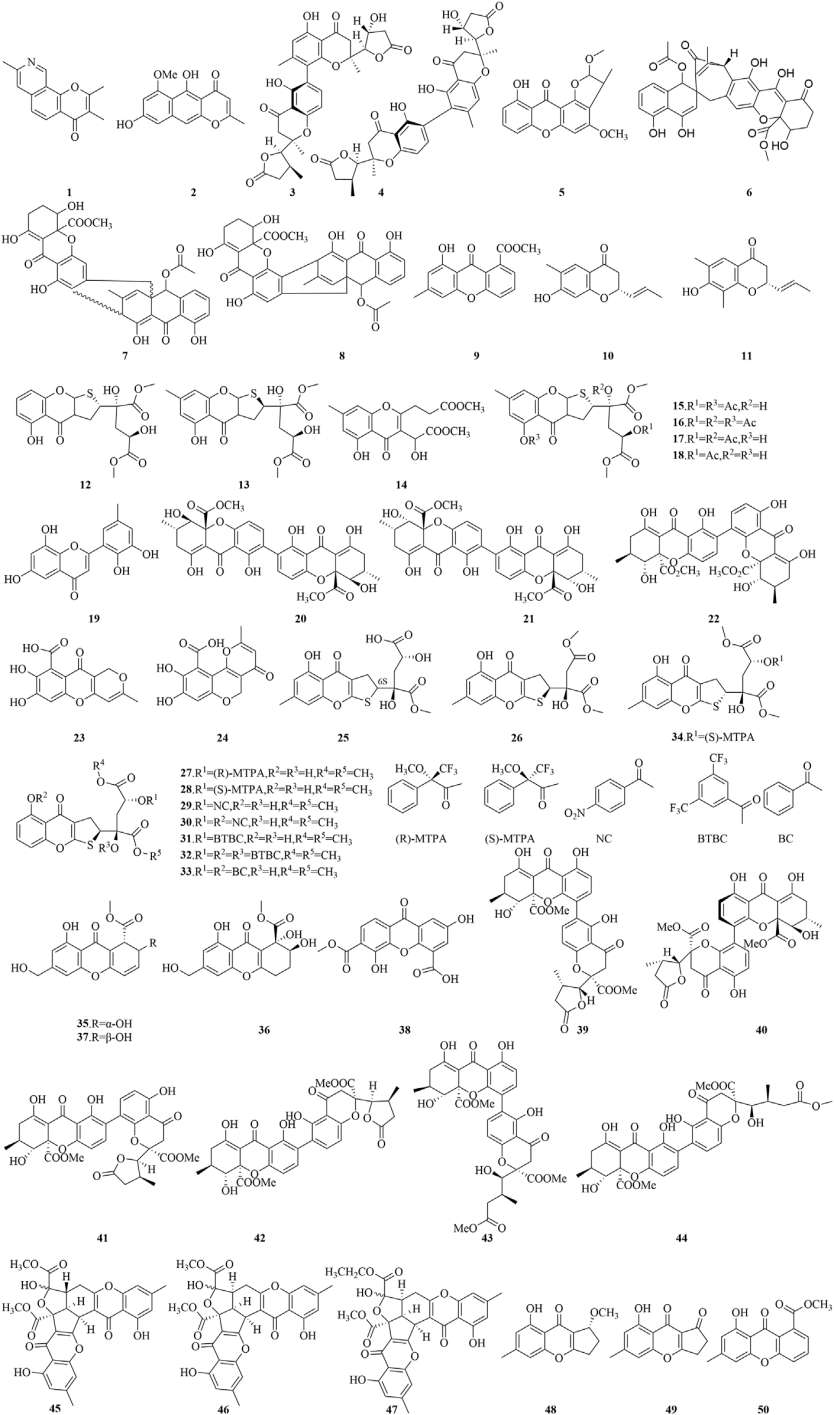
Structural formulas of the isolated compounds **1–50**.

**FIGURE 6:**
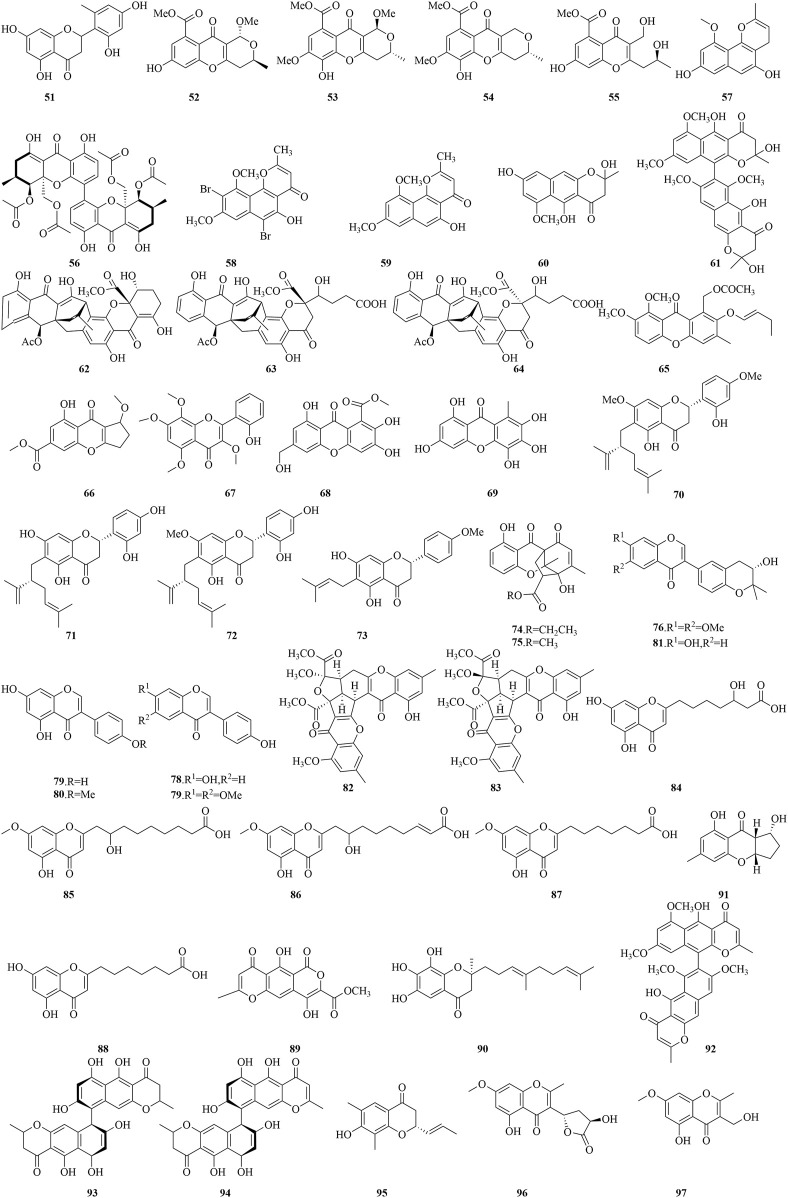
Structural formulas of the isolated compounds **51–97**.

Chromones (4H-1-benzopyran-4-ones) represent a significant class of oxygen-containing heterocycles with a benzo-γ-pyrone skeleton (Duan et al., 2019). Not only are these compounds abundant and structurally complex, but they have also been demonstrated to be a unique skeleton structure in medicinal chemistry, exhibiting a broad spectrum of biological properties, encompassing antibacterial, anticancer, anti-inflammatory, and antioxidant activities. The biological potential of this skeleton structure, coupled with its minimal toxicity to mammals, have spurred the advancement of a variety of chromone-based pharmaceuticals, highlighted by Chromonar, Flavoxate, and Scutellarein (Silva et al., 2018). The structures of chromone compounds derived from marine fungi are depicted in Figures 5, 6.

Aspergillitine (**1**), a chromone derivative with a pyridine ring, isolated from the sponge-associated fungus *Aspergillus versicolor*, has demonstrated antibacterial properties against *Bacillus subtilis*, achieving an inhibition zone diameter of 7 mm at 5 μg and 8 mm at 10 μg (Lin et al., 2003). The chromone TMC-256A1 (**2**), derived from the culture of *Aspergillus niger*, displayed significant antiviral activity (Ovenden et al., 2004). Notably, compound **2** efficaciously inhibited the translational activity mediated by the internal ribosome entry site (IRES), with an IC_50_ value of 44 μM. Furthermore, this compound underwent a selectivity assay, targeting the inhibition of both viral and mammalian Cap-dependent translation initiation pathways, exhibiting an IC_50_ of 80 μM. Two dimeric chromone derivatives, monodictyochromes A and B (**3**, **4**), were isolated from the marine-derived fungus *Monodictys putredinis* (Pontius et al., 2008), have shown significant inhibitory effects on the activity of the cytochrome P450 isoenzyme CYP 1A, with IC_50_ values of 5.3 and 7.5 μM, respectively. While compound **3** showed slightly diminished potency in inhibiting aromatase activity in comparison to compound **4**, with IC_50_ values of 24.4 and 16.5 μM, respectively. Additionally, compounds **3** and **4** have shown notable potency as activators of NNAD(P)H: quinone oxidoreductase (NQO1), achieving CD values of 22.1 and 24.8 μM, which represent the concentrations doubling the enzyme’s specific activity. Collectively, these findings suggest that compounds **3** and **4** are promising candidates for chemoprevention strategies, as they attenuate the activity of cytochrome P450 enzymes implicated in carcinogenesis and robustly induce NQO1 activity, thereby potentially enhancing the body’s detoxification mechanisms against carcinogens.

Asperxanthone (**5**), extracted from the marine-derived fungus *Aspergillus* sp. MF-93, sourced from the Quanzhou Bay, has been found to exhibit a moderate inhibitory effect on the tobacco mosaic virus (TMV) proliferation, with a 62.9% inhibition rate observed at a 0.2 mg/mL concentration (Wu et al., 2009). The sponge-derived fungus *Tritirachium* sp. SpB081112MEf2 has yielded compounds JBIR-97 (**6**), JBIR-98 (**7**), and JBIR-99 (**8**), which have potently inhibited the proliferation of human cervical carcinoma HeLa cells, with IC_50_ values of 11, 17, and 17 μM, respectively, and against human malignant pleural mesothelioma ACC-MESO-1 cells with IC_50_ values of 31, 63, and 59 μM, respectively (Ueda et al., 2010). Compound **9**, a newly identified mutant janthinone, extracted from the marine fungus *Penicillium purpurogenum* G59, has shown notable inhibitory activity against the K562 human chronic myelogenous leukemia cell line at a 100 μg/mL concentration, achieving a 34.6% inhibition rate (Chai et al., 2012). Two chromone ketones (**10**, **11**), isolated from *Trichoderma* sp., exhibited significant proliferation inhibitory activity against the human breast cancer cell line MCF-7, with IC_50_ values of 7.82 and 9.51 μg/mL, respectively (Lan et al., 2012). Additionally, the novel oxalicumones A-C (**12**-**14**), along with four known compounds **15**-**18,** were isolated from marine-derived fungus *Penicillium macum* SCSGAF 0023 (Sun et al., 2012). All these compounds exhibited cytotoxic effects against various human cancer cell lines such as melanoma A375, lung carcinoma A549, cervical carcinoma HeLa, liver hepatocellular carcinoma HepG2, colonic adenocarcinoma SW-620, as well as the normal liver L-02 cell line. Specifically, compound 12 displayed moderate cytotoxicity against A375 and SW-620, with IC_50_ values at 11.7 µM and 22.6 µM. Compounds **13** and **16** showed mild cytotoxic effects in these cell lines, with IC_50_ values ranging from 27.8 to 42.7 µM. In contrast, compound **14** exhibited no cytotoxic effects on any of the cell lines evaluated. Analysis of the structure-activity relationship indicated that incorporating a 2,3-dihydrothienyl group markedly enhances the cytotoxic efficacy of chromone-based compounds. Moreover, the presence of a bulky substituent at the OH-13 position appears to augment cytotoxicity, whereas acetylation at the C-1, C-11, and/or C-13 positions appears to mitigate it, underscoring the importance of structural features in dictating biological activity.

A novel polyketide, identified as 6,8,5′6′-tetrahydroxy-3′-methylflavone (**19**), along with the analogues secalonic acid D (**20**), secalonic acid B (**21**), and penicillixanthone A (**22**), has been extracted from the fermentation products of the gorgonian-associated fungus *Penicillium* sp. SCSGAF 0023 (Bao et al., 2013). In antibacterial assays, compounds **19**–**22** demonstrated discernible inhibitory effects against four bacterial strains tested (*B. subtilis*, *Escherichia coli* JVC1228, *Micrococcus luteus* UST950701-006, *Pseudomonas nigritaciens* UST010620-005), exhibiting MIC values that varied from 24.4 to 390.5 μg/mL. An in-depth investigation into the antibiofilm potential of compound **20** against *Staphylococcus oneidensis* MR-1 revealed its efficacy in completely inhibiting biofilm formation at a concentration of 3.125 mg/mL following a 12-h incubation period. Furthermore, in antifouling assays, compound **19** significantly impeded the settlement of *Balanus amphitrite* larvae, with an EC_50_ value of 6.71 mg/mL. The marine fungus Penicillium sp. JF-55 has yielded two bioactive secondary metabolites: anhydrofulvic acid (**23**) and citromycetin (**24**). Compound **23** demonstrated potent, concentration-dependent inhibition of Protein tyrosine phosphatase 1B (PTP1B), with an IC_50_ value of 1.90 μM. Compound **24**, however, showed no significant inhibitory activity against PTP1B. These findings suggest that the linear tricyclic framework and the strategic positioning of the carbonyl group in compound **23** are pivotal structural features that enhance its binding affinity and interaction with the PTP1B active site, highlighting the importance of molecular architecture in modulating biological activity (Lee et al., 2013).

In addition, the secondary metabolites of the gorgonian-associated fungus *Penicillium oxalicum* SCSGAF 0023 have yielded four dihydrothieno-chromene derivatives, specifically pecicumones A and B (**12**-**13**) along with pecicumones D and E (**25**-**26**). Through acylation reactions of compounds **12** and **13**, eight derivatives (**27**-**34)** were synthesized. Significantly, compounds **12**, **26**, and **29** demonstrated potent cytotoxicity against a selection of 8 cell lines (H1975, U937, K562, BGC823, MOLT-4, MCF-7, HL60, Huh-7), achieving IC_50_ values below 10 μM. Compound **13** was found to exert cytotoxic effects on the U937 and MOLT-4 cell lines, achieving IC_50_ values of 5.0 μM and 2.30 μM, respectively. Meanwhile, compound **25** also displayed cytotoxicity against the BGC823 and MOLT-4 cell lines, yielding IC_50_ values of 10.10 and 5.74 μM, respectively. Compound **27** demonstrated cytotoxic activity against U937, BGC823, and MOLT-4 cell lines with IC_50_ values of 9.85, 8.82, and 5.89 μM, respectively. Compound **28** demonstrated potent cytotoxic effects against a spectrum of cell lines, including U937, K562, BGC823, MOLT-4, and HL60, with IC_50_ values spanning from 1.34 to 9.04 μM. Compounds **30**-**31** also exhibited significant cytotoxicity against a broader panel of cell lines (U937, K562, BGC823, MOLT-4, MCF-7, and HL60), with IC_50_ values between 1.04 and 6.94 μM. Furthermore, compounds **33**–**34** showed cytotoxic activity specifically against MOLT-4 and HL60 cell lines, with IC_50_ values ranging from 3.99 to 9.14 μM. The structure-activity relationship analysis indicates that the 2,3-dihydrothieno structural motif is a principal determinant of cytotoxicity for this class of compounds. Furthermore, the presence of the 1-OH group and the two O-methyl groups at carbons 16 and 17 significantly enhance their biological potency, while the 13-OH group seems to be non-essential for the cytotoxicity of compound **12**. Comparative cytotoxicity studies between compounds **12** and **25**, and **28** and **34**, have disclosed that the absolute configuration at carbon 6 influences the cytotoxicity of compound **12**, underscoring the importance of stereochemistry in modulating the biological activity of these marine-derived natural products (Bao et al., 2014).

The deep-sea fungus *Engyodontium album* DFFSCS 021 yielded Engyodontiumone H (**35**) and two associated polyketide compounds (**36**-**37**). Notably, compounds **35** and **37** exhibited significant cytotoxic effects on the U937 cell line, achieving IC_50_ values below 10 μM. They also exhibited moderate antibacterial properties against *E. coli* and *B. subtilis*, with MIC values not exceeding 64.0 μg/mL. Moreover, compound **36** has been identified as a potent antifoulant, significantly inhibiting the settlement of barnacle *B. amphitrite* larvae at an EC_50_ of 19.1 μg/mL, while maintaining a favorable safety profile (Yao et al., 2014). Additionally, the mangrove-associated fungus *Penicillium chrysogenum* HND11-24 has yielded an oxanthrone derivative, penixanacid A (**38**), which has shown a spectrum of bioactivities against cancer cell lines such as HeLa, BEL-7402, HEK-293, HCT-116, and A549, with IC_50_ values in the range of 10–30.6 μM (Guo et al., 2015). Six flavone-chromone dimers, versixanthones A-F (**39**-**44**) and compound **20**, were isolated from the cultured extracts of the mangrove-associated fungus *A. versicolor* HDN1009. Compounds **39**–**41** displayed significant selective cytotoxic effects against the HL-60 and K562 cell lines, with IC_50_ values ranging from 2.6 to 18.2 μM. Furthermore, compounds **20** and **42**–**44** demonstrated broad cytotoxicity against seven cancer cell lines (HL-60, K562, A549, H1975, 803, HO-8910, HCT-116) with IC_50_ values between 0.7 and 14.0 μM (Wu et al., 2015). From the diethyl sulfate (DES)-treated mutant strain of *P. purpurogenum* G59, a group of compounds, epiremisporine B (**45**), epiremisporine B1 (**46**), isoconiochaetone C (**47**), remisporine B (**48**), coniochaetone A (**49**), and methyl 8-hydroxy-6-methyl-9-oxo-9H-xanthene-1-carboxylate (**50**), was isolated. At a concentration of 100 μg/mL, they demonstrated substantial anticancer effects against K562, HL-60, HeLa, and BGC-823 cell lines, resulting in inhibition rates (IR%) between 11.4% and 80.0%. Specifically, compounds **45**–**47** showed IC_50_ values between 53.1 and 83.1 μg/mL against the K562 and HL-60 cell lines (Xia et al., 2015).

Penimethavone A (**51**), a novel flavonoid, was isolated from the fungus *P. chrysogenum*, which is symbiotically associated with the coral *Carijoa* sp. from the South China Sea. This compound is characterized by a distinctive methyl group on its B-ring and exhibited potent cytotoxicity against HeLa and rhabdomyosarcoma (RD) cell lines, with IC_50_ values of 8.41 μM and 8.18 μM, respectively (Hou et al., 2016). Phomopsichins A-D (**52**-**55**) and the known phomoxanthone A (**56**) were extracted from the mangrove-associated endophytic fungus *Phomopsis* sp. 33#. They showed a slight inhibitory effect on AChE and α-glucosidase, with inhibition rates from 2.7% to 38.4% at 250 μM. Moreover, they exhibited a moderate ability to scavenge free radicals, including DPPH and OH radicals, with inhibition rates of 3.5%–40.0% at 1 mM (Huang et al., 2016b). TMC-256C1 (**57**) isolated from the extract of the marine-derived fungus *Aspergillus* sp. SF6354, has been shown to confer resistance to hippocampal HT22 cells against kainic acid-induced cytotoxicity and oxidative stress mediated by reactive oxygen species (ROS). Additionally, it exerts inhibitory effects on inflammatory processes triggered by lipopolysaccharide (LPS) in BV2 cells. The neuroprotective and anti-neuroinflammatory efficacies of compound **57** are attributed to the upregulation of heme oxygenase (HO)-1 and nuclear factor-E2-related factor 2 (Nrf2), which translocate to the nucleus in both HT22 and BV2 cell lines. It is further suggested that compound **57** may promote the nuclear accumulation of Nrf2 and the subsequent activation of HO-1 protein expression via the engagement of p38 mitogen-activated protein kinase (MAPK) and phosphatidylinositol-3 kinase (PI3K)/protein kinase B (Akt) signaling pathways (Kim et al., 2016).

In an innovativel bioprocess optimization approach, metal bromides were introduced to the fermentation of the marine mudflat fungus *A. niger*, resulting in the production of a potent free radical scavenger, 6,9-di-bromoflavasperone (**58**), along with three naphthopyranone monomers, TMC-256A1 (**2**), flavasperone (**59**), and fonsecin (**60**), as well as a naphthopyranone dimer, aurasperone B (**61**). These compounds demonstrated significant free radical scavenging capabilities against the DPPH radical, with IC_50_ values ranging from 0.01 to 25 μM (Leutou et al., 2016). This study underscores the importance aromatic hydroxyl group count in determining the free radical scavenging efficacy, highlighting that an increased number of these groups significantly enhances the compounds' antioxidant potential. An anthraquinone-xanthone polyketide compound (**8**) with an alkaloid-free structure, along with three additional polyketide compounds (**62**-**64**), isolated from the fungus *E. album* strain LF069, exhibited potent inhibitory effects against *S. epidermidis* and methicillin-resistant *Staphylococcus aureus* (MRSA), with IC_50_ values ranging from 0.21 to 6.77 μM and 0.22–3.41 μM, respectively. Moreover, compounds **8** and **62** also exhibited modest cytotoxic effects on the murine fibroblast cell line NIH3T3, with IC_50_ values of approximately 13 μM (Wu et al., 2016).

Versicones G (**65**), a flavonoid compound isolated from the mangrove-associated *A. versicolor* strain HDN11-84, has demonstrated significant cytotoxicity against Hela, HL-60, and NB4 cell lines, with IC_50_ values between 15.6 and 21.7 μM (Li et al., 2017). Meanwhile, Epiremisporine B (**46**) and Coniochaetone J (**66**), from the deep-sea sediment-derived *Penicillium* SCSIO Ind16F01, have shown weak inhibitory activity against EV71 *in vitro*, with IC_50_ values of 19.8 and 81.6 µM, respectively. Additionally, compound 66 has displayed inhibitory activity against the H3N2 virus, with an IC_50_ value of 24.1 µM, and cytotoxic effects on cancer cell lines K562, MCF-7, and SGC7901, with IC_50_ values of 16.6, 16.3, and 15.8 µM, respectively (Liu et al., 2017). Derived from the gorgonian coral *Anthogorgia ochracea* of the South China Sea, *Aspergillus candidus* has yielded Aspergivones B (**67**), which has shown a modest inhibitory effect on α-glucosidase, achieving an IC_50_ value of 244 μg/mL (Ma et al., 2017). Arthone C (**68**) and compound (**69**), extracted from the deep-sea fungus *Arthrinium* sp. UJNMF0008, displayed remarkable antioxidant activity, with IC_50_ values for DPPH radical scavenging of 16.9 and 22.1 μM, respectively, and for ABTS radical scavenging of 18.7 μM and 18.0 μM, respectively (Bao et al., 2018). From the fermentation of *Streptomyces* sp. G248, two new flavonoids (**70**-**71**) and two existing compounds (72-73) were identified. Compounds **70** and **71** exhibited pronounced inhibitory activities against the bacterial strains tested (*Enterococcus faecalis*, *S. aureus*, *Bacillus cereus*, *Pseudomonas aeruginosa*, *Salmonella enterica*, *Candida albicans*), with IC_90_ values from 1 to 16 μg/mL. Furthermore, compounds 72 and 73 have also shown considerable anti-tuberculosis activity, with IC_90_ values of 6.0 μg/mL and 11.1 μg/mL against *Mycobacterium tuberculosis* H37Rv, respectively. The methoxy group at position C-7 and the hydroxyl group at C-4 are considered crucial for their anti-tuberculosis activity, given the lack of effect from compounds **70** and **71** on *M. tuberculosis*. Additionally, cytotoxicity assays revealed that compounds **70** and **72** possess inhibitory effects on four tested cancer cell lines (KB, Hep-G2, Lu-1, and MCF-7), with IC_50_ values ranging from 2.0 to 14.6 μg/mL (Cao et al., 2019).

Two polycyclic chromone compounds, penixanthones C-D (**74**-**75**), isolated from the fungus *Penicillium* sp. SCSIO 041218, sourced from mangrove sediments, showed considerable inhibitory effects on the proliferation of K562, MCF-7, and Huh-7 cells, with IC_50_ values ranging from 55.2 to 67.5 μM (Huang et al., 2019). The marine-derived fungus *Aspergillus terreus* C23-3, cultivated on a soybean substrate, has yielded six isoflavone derivatives (**76**-**81**) and have exhibited potent anti-inflammatory activity in RAW 264.7 macrophages stimulated by LPS, achieving a 36% inhibition rate at 32 μM, without exhibiting cytotoxic effects. Furthermore, compounds **84**–**81** showed broad-spectrum antimicrobial efficacies against both Gram-positive and Gram-negative bacteria, as well as antifungal activity, with inhibition zones of 10.0–13.8 mm at a disk concentration of 10 μg. Compound **77** displayed moderate inhibition of AChE, with an IC_50_ of 42.5 μM. Additionally, compounds **77**, **80**, and **81** demonstrated potent lethal effects on brine shrimp larvae, with LC_50_ values ranging from 11.6 to 68.2 μM, substantially lower than that of the positive control Hg(NO_3_)_2_, which had an LC_50_ of 77.0 μM, suggesting their utility in the development of novel pesticides (Yang et al., 2020a).

Isolated from the marine-derived fungus *Penicillium citrinum*, two novel chromone derivatives, epiremisporine D (**82**) and E (**83**), along with the known compound epiremisporine B (**46**), have been found to markedly decrease the production of superoxide anions in human neutrophils stimulated by formyl-L-methionyl-L-leucyl-L-phenylalanine (fMLP), with IC_50_ values of 6.39, 8.28, and 3.62 µM. Moreover, compounds **46** and **83** have displayed cytotoxic activity against A549 cells, with IC_50_ values of 32.29 µM and 43.82 µM. Compounds **82** and **83** have also been identified to induce apoptosis in A549 cells, engaging the mitochondrial pathway and a caspase 3-dependent process (Chu et al., 2021). Three novel chromone derivatives, penithochromones R-T (**84**-**86**), along with the known compounds **87** and **88**, were isolated from the fungus *Penicillium thomii* YPGA3, sourced from the sediments of the Yap Trench. These compounds showed notable inhibitory activity against α-glucosidase, with IC_50_ values ranging from 268 to 1,017 μM, while higher than that of the positive control drug acarbose at 1.3 μM (Han et al., 2021). Furthermore, the marine fungus *Taeniolella* sp. BCC31839 has yielded compound **89**, which has exhibited promising antimalarial properties with an IC_50_ of 9.75 μg/mL. Additionally, this compound has displayed antibacterial properties against *B. cereus*, with a MIC of 12.5 μg/mL, and has exhibited moderate cytotoxic effects on NCI-H187 cells, resulting in an IC_50_ of 23.66 μg/mL (Intaraudom et al., 2021). Pestalotiochromones A (**90**) was isolated from the marine-derived algal fungus *Pesacetalotiopsis neglecta* SCSIO 41403. Surface plasmon resonance (SPR) analysis revealed that compound **90** exhibited a high binding affinity for Liver X receptors (LXRs), with a dissociation equilibrium constant (KD) of 6.2 μM, suggesting that compound **90** may represent a novel LXR agonist (Liang et al., 2021). Aspergilluone A (**91**), obtained from the marine fungus *Aspergillus* sp. LS57, has demonstrated considerable *in vitro* activity against *M. tuberculosis*, reaching a MIC value of 32 μg/mL. It has also manifested moderate antibacterial effects against *S. aureus*, with a MIC of 64 μg/mL. However, its activity against *B. subtilis* and *E. coli* was less pronounced, both showing MIC values of 128 μg/mL (Liu et al., 2021). Aurasperone A (**92**), identified from the marine *A. niger*, has proven to be a potent inhibitor of SARS-CoV-2, with an IC_50_ value of 12.25 μM, and has also shown remarkably low cytotoxicity to Vero E6 cells, as indicated by a CC_50_ value of 32.36 μM and a selectivity index (SI) of 2,641.5 (ElNaggar et al., 2022).

A chemical investigation was conducted on the co-culture of the marine fungus *Cosmospora* sp. with the plant pathogen Magnaporthe oryza, resulting in the isolation of two chromones, Cephalochromin (**93**) and Ustilaginoidin G (**94**). Compound **93** displayed potent inhibitory effects against *Phytophthora infestans*, *Xanthomonas campestris*, and *Ralstonia solanacearum*, with IC_50_ values of 2.3, 27.6, and 12.1 μg/mL, respectively. While compound **94** has proven to have a moderate inhibitory effect against *P. infestans* and *X. campestris*, with IC_50_ values of 7.2 μg/mL and 21.7 μg/mL, respectively (Oppong-Danquah et al., 2022). Moreover, compound **95**, sourced from the marine fungus *P. citrinum* VM6, has displayed a more pronounced antibacterial effect on Gram-positive bacteria and yeasts compared to Gram-negative bacteria, with MIC values of 128, 64, 128, 256, and 16 μg/mL against *E. faecalis*, *S. aureus*, *B. cereus*, *S. enterica*, and *C. albicans*, respectively (Anh et al., 2023). Extracted from the mangrove-inhabiting fungus *Trichoderma lentiforme* ML-P8-2, compounds **96** and **97** have exhibited a limited cytotoxic impact on A549 cells, with an IC_50_ value of 47.2 μM. Compound **97** has also demonstrated a moderate inhibitory effect against *C. albicans*, reaching a MIC value of 25 µM. In addition, both compounds **96** and **97** have shown some inhibitory activity against AChE, with IC_50_ values of 38.6 µM and 20.6 µM, respectively (Yin et al., 2023).

### 4.2 Isocoumarin

**FIGURE 7:**
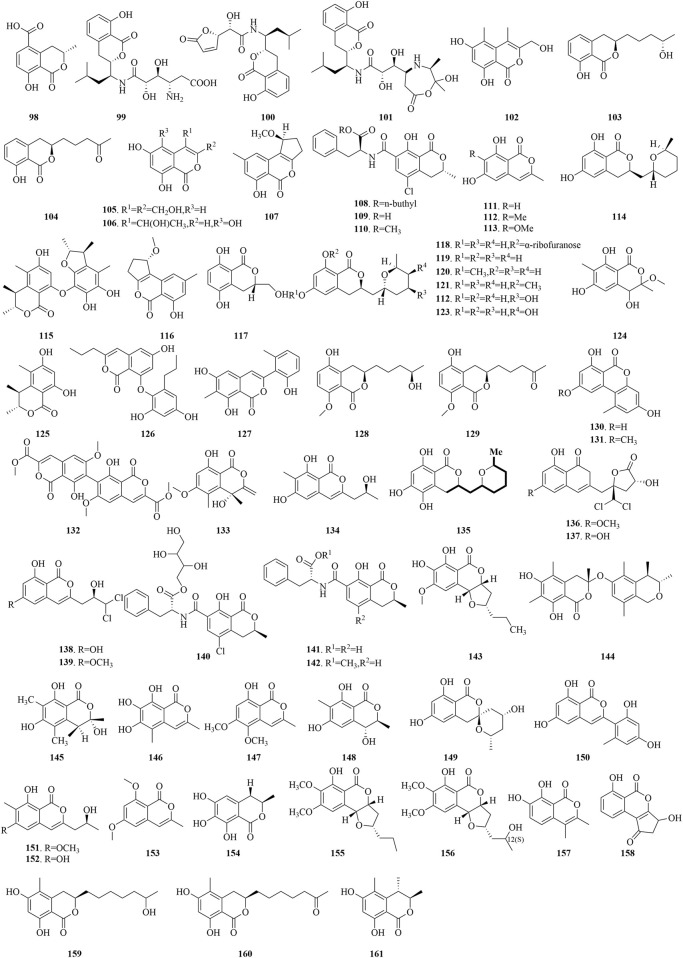
Structural formulas of the isolated compounds **98–161**.

The isocoumarin skeleton, chemically defined as 3,4-dihydro-2H-chromen-2-one, represents a pivotal class of O-heterocyclic compounds (Ji Ram et al., 2014). It serves not only as the backbone for a multitude of natural products but also demonstrates immense promise in the realm of drug discovery. The widespread occurrence and diversity of these compounds in nature, coupled with the array of bioactivities exhibited by their derivatives, such as antibacterial, anti-inflammatory, anticancer, and protease inhibitory properties, have propelled them to the forefront of pharmaceutical research. Isocoumarins derived from marine fungi, as illustrated in Figure 7, are particularly noteworthy for their unique chemical structures and potential therapeutic applications.

The marine fungus *Halorosellinia oceanica* BCC 5149 has yielded 5-carboxymellein (**98**), a secondary metabolite that has demonstrated cytotoxic effects against the KB and BC-1 cell lines, achieving IC_50_ values of 3 μg/mL. Moreover, this compound has also displayed antimalarial properties, with an IC_50_ of 4 μg/mL (Chinworrungsee et al., 2001). Three isocoumarins, AI-77-B (**99**), AI-77-F (**100**), and Sg17-1-4 (**101**), were isolated from the marine fungus *Alternaria tenuis* Sg17-1. Notably, compound **99** has exhibited potent suppression of cell proliferation in A375-S2 and Hela cells, with IC_50_ values of 0.1 mM and 0.02 mM, respectively. Compound **101** follows with moderate inhibitory effects, having IC_50_ values of 0.3 mM and 0.05 mM for the aforementioned cell lines. Conversely, compound **100** displays relatively low activity against Hela cells, indicated by a higher IC_50_ value of 0.4 mM (Huang et al., 2006). Furthermore, from the marine fungus *Arthrinium sacchari*, decarboxyhydroxycitrinone (**102**) has been isolated and has shown significant inhibition of proliferation in human umbilical vein endothelial cells (HUVECs) and human umbilical artery endothelial cells (HUAECs), with IC_50_ values of 7.6 and 17.4 μM, respectively (Tsukada et al., 2011). Penicimarins F (**105**) and three isocoumarin derivatives (**103**, **104**, **106**), sourced from the sponge-derived *Penicillium* sp. MWZ14-4 of the South China Sea. Compounds **103** and **104** have shown certain antibacterial effects against *Staphylococcus albus*, with MIC values of 12.5 μM. Compound 105 has demonstrated certain antibacterial activity against *S. aureus*, with a MIC value of 12.5 μM. Meanwhile, compound 106 has shown moderate activity against *B. subtilis* and *Vibrio parahemolyticus*, with MIC values of 6.25 μM (Qi et al., 2013). Derived from the marine fungus *Penicillium* sp. ML226, compound **107** has demonstrated low cytotoxicity against HeLa and HepG-2 cell lines at a concentration of 10 μg/mL, resulting in inhibition rates of 4% and 16.1%, respectively. Additionally, ochratoxin A n-butyl ester (**108**), ochratoxin A (**109**), and ochratoxin A methyl ester (**110**), extracted from the fermentation products of the marine fungus *Aspergillus* sp.SCSGAF0093, have exhibited considerable toxicity to brine shrimp, with LC_50_ values of 4.14, 13.74, and 2.59 μM, respectively (Xu et al., 2013).

The sponge-associated fungus *Aspergillus similanensis* KUFA 0013 has yielded a set of compounds, including 6,8-dihydroxy-3-methylisocoumarin (**111**), 6,8-dihydroxy-3,7- dimethylisocoumarin (**112**), and reticulol (**113**), which have shown moderate antibacterial activity against ampicillin-resistant bacteria, with inhibition zones of 2.5–5 mm at a concentration of 15 μg/disc (Prompanya et al., 2014). Asperentin (**114**), obtained from the marine fungal *Aspergillus* sp. F00785, has exhibited inhibitory effects against *Colletotrichum gleosporioides Penz*, *C. gleosporioides (Penz.) Sacc*, and *Botrytis cinerea Pers* at a 5 mg/mL concentration, with inhibition diameters of 19.7, 13.3, and 1.67 mm, respectively (Tang et al., 2014). Penicitol B (**115**) and Penicitol C (**116**), identified from the mangrove-associated *P. chrysogenum* HND 11-24, have demonstrated diverse cytotoxic effects against a panel of cell lines including HeLa, BEL-7402, HEK-293, HCT-116, and A-549, with IC_50_ ranges of 3.4–9.6 μM and 10.8–40.5 μM, respectively (Guo et al., 2015). Compound **117**, isolated from the endophytic fungus *Botryosphaeria* sp. KcF6, derived from the mangrove plant *Kandelia candel*, has displayed significant inhibitory activity against cyclooxygenase-2 (COX-2), with an IC_50_ value of 6.51 μM (Ju et al., 2015). From the marine-derived fungi *Aspergillus* sp. SF-5974 and *Aspergillus* sp. SF-5976, a series of compounds (**118**-**123**) has been isolated, exhibiting potent inhibitory effects on the LPS-induced production of nitric oxide (NO) and prostaglandin E2 (PGE2) in BV2 microglia and presented IC_50_ ranges of 20–65 μM and 21–61 μM, respectively. Of particular interest, compound **118** was observed to influence inflammatory pathways, reducing the phosphorylation of IκB-α, impeding NF-κB nuclear translocation, and lowering p38 MAPK activation, suggesting its anti-inflammatory potential (Kim et al., 2015). Compound **124**, sourced from the mangrove endophytic *Aspergillus* sp. 16-5B and grown in Czapek medium, has been found to inhibit α-glucosidase activity, with an IC_50_ value of 90.4 μM (Liu et al., 2015).

Decarboxydihydrocitrinone (**125**), extracted from the co-culture of the marine fungi *P. citrinum* and *Beauveria felina,* has exhibited modest inhibitory effects against *Vibrio alginolyticus*, with a MIC value of 8.0 μg/mL (Meng et al., 2015). Spiromastols I (**126**), discovered from the deep-sea fungus *Spiromastix* sp. MCCC3A00308, has demonstrated moderate antibacterial activity against a range of bacterial strains, including *Xanthomonas vesicatoria*, *Pseudomonas lachrymans*, *Agrobacterium tumefaciens*, *R. solanacearum*, *Bacillus thuringiensis*, *S. aureus*, and *B. subtilis*, with MIC values falling between 8 and 16 μg/mL (Niu et al., 2015). Pleosporalone A (**127**), identified from the Bohai Sea sediment-derived *Pleosporales* sp. CF09-1, has displayed significant inhibitory effects against three phytopathogenic fungi, *B. cinerea*, *Rhizopus sticarum*, and *Phytophthora capsici*, with MIC values of 0.39, 0.78, and 0.78 μM, respectively (Cao et al., 2016). From the mangrove-associated *P. citrinum* of the South China Sea, compounds **128** and **129** have been extracted and have shown significant antibacterial activity against a panel of pathogenic bacteria (*Staphylococcus epidermidis*, *S. aureus*, *E. coli*, *B. cereus*, and *V. alginolyticus)* with MIC values from 10 to 20 μM (Huang et al., 2016a). Alternariol (**130**) and alternariol-9-methylether (**131**), obtained from the fungus *Aspergillus* sp. and *Botryotinia fuckeliana* of the Wadden Sea in Germany, have been identified as inhibitors of the glycogen synthase kinase-3β (GSK-3β) enzyme, with IC_50_ values of 0.13 μM and 0.20 μM, respectively (Wiese et al., 2016). A novel dimeric isocoumarin, bipenicilisorin (**132**), extracted from the deep-sea derived fungus *P. chrysogenum* SCSIO 41001, has exhibited pronounced cytotoxic effects on the K562, A549, and Huh-7 cell lines, with IC_50_ values of 6.78, 6.94 μM, and 2.59 μM, respectively (Chen et al., 2017). Moreover, The deep-sea sediment-associated fungus *Leptosphaeria* sp. SCSIO 41005 has yielded compound **133**, which has proven effective in inhibiting the growth of *Cochliobolus miyabeanus*, reaching an IC_50_ value of 0.5 μM. It has also shown a limited inhibitory impact on the formation of *C. albicans* biofilms, with a MIC value of 101 μM (Luo et al., 2017).

Cultivation of the mangrove-derived *Eurotium chevalieri* KUFA 0006 has led to the discovery of compound **134**, which has displayed a remarkable efficacy in reducing *E. coli* biofilms. In addition to its standalone effects, when co-administered with cefotaxime (CTX), compound **134** exhibits a subtle synergistic action. Furthermore, the compound has been observed to induce a weak or moderate enlargement of the partial inhibition zone surrounding vancomycin (VAN) in vancomycin-resistant *E. faecalis* (VRE) B3/101, enhancing the inhibitory effect compared to the application of VAN alone (May Zin et al., 2017). Asperentin B (**135**), isolated from the fungus *Aspergillus sydowii*, derived from the deep-sea sediment of the Mediterranean, has exhibited a robust inhibition of protein tyrosine phosphatase 1B (PTP1B) activity, with an IC_50_ value of 2.05 μM, outperforming the positive control suramin by sixfold (Wiese et al., 2017). Isolated from the *Ascomycota* sp. CYSK-4, an endophyte of mangroves, dichloro-diaportintone (**136**), desmethyldichlorodiaportintone (**137**), and two analogues (**138**-**139**) have demonstrated the ability to inhibit the production of NO in LPS-induced RAW 264.7 murine macrophages, displaying IC_50_ values from 15.8 to 67.2 μM. Additionally, compounds **138** and **139** have shown significant antibacterial potency against several bacterial strains, including *S. aureus*, *B. subtilis*, *E. coli*, *Klebsiella pneumoniae*, and *Acinetobacter calcoaceticus*, with MIC values ranging from 25 to 50 μg/mL. Compound **136** showed MIC values of 50 μg/mL against *S. aureus*, *E. coli*, and *K. pneumoniae*. Notably, a comparative analysis revealed that compounds **137** and **138**, which bear a hydroxyl group at the C-6 position, manifest stronger anti-inflammatory effects than compounds **136** and **139**, which contain a methoxy group at the same position (Chen et al., 2018). This observation underscores the influential role of the C-6 substituent on the anti-inflammatory properties of these compounds.

Ochratoxin A_1_ (**140**) and its two analogues (**141**-**142**), obtained from the sponge-associated *Aspergillus ochraceopetaliformis*, have exhibited considerable inhibitory effects on LPS-induced interleukin-6 (IL-6) and tumor necrosis factor-alpha (TNF-α) expression in THP-1 cells at 10 μM, achieving inhibition rates of 74.4%–91.6% for IL-6 and 67.7%–72.9% for TNF-α. Importantly, these compounds were confirmed to be non-cytotoxic to THP-1 cells (Liu et al., 2018). Moreover, compound **143**, from the sponge-derived *Setosphaeria* sp. SCSIO 41009, has shown robust DPPH radical scavenging activity, with an IC_50_ value of 38 μM (Pang et al., 2018b). Penicitol D (**144**) and an associated compound **145**, isolated from the deep-sea fungus *P. citrinum* NLG-S01-P1, have demonstrated potent activity. Compound **144** has manifested significant cytotoxicity against HeLa cells, with an IC_50_ value of 4.1 μM. Moreover, Compound **144** has shown potent activity against MRSA strains with an MIC range of 7–8 μg/mL, while compound **145** has exhibited strong antimicrobial effects against *Vibrio vulnificus* and *Vibrio campbellii*, with MIC values in the range of 15–17 μg/mL (Wang et al., 2019). Additionally, compound **145** has shown moderate inhibitory activity against both the standard strain G27 and the clinically relevant drug-resistant strain HP159 of *Helicobacter pylori*, with MIC values of 16 μg/mL (Lai et al., 2023). The marine mangrove endophytic fungus *Botryosphaeria ramosa* L29 has yielded Botryospyrones A-C (**146**-**148**), which have been assessed for their *in vitro* antifungal capabilities against a selection of phytopathogenic fungi. Compound **146** has shown promising antimicrobial potency against *Fusarium oxysporum* with an MIC of 112.6 μM and moderate efficacy against *Penicillium italicum* with an MIC of 450.4 μM. Compound **147** has exhibited MIC values of 105.8 μM against *F. oxysporum*, and 211.7 μM against both *P. italicum* and *Fusarium graminearum*. Compound **148** has demonstrated moderate activity against *F. oxysporum* and *F. graminearum*, with MIC values of 223 μM for both (Wu et al., 2019). Demethylcitreoviranol (**149**), isolated from *Penicillium* sp. KMM 4672, has shown interesting bioactivity profiles. Interestingly, compound **149** exhibited neutrality towards the viability of Neuro-2a cells when exposed to 6-hydroxydopamine (6-OHDA), it exerted a pronounced protective effect within cellular models of Parkinson’s disease induced by paraquat (PQ) and rotenone. Specifically, in PQ-induced scenarios that reduced cell viability by 44%, compound **149** augmented the survival of Neuro-2a cells, elevating viability by 34.3%. Similarly, in the presence of rotenone, which caused a 48% decline in cell viability, compound **149** significantly improved cell survival, increasing viability by 65.2%. Expanding the investigation to the influence of the compound on oxidative stress, it was observed that 6-OHDA, PQ, and rotenone escalated intracellular ROS levels by 66%, 48%, and 79%, respectively. Notably, compound **149** effectively mitigated the ROS increase, observed specifically in the PQ- and rotenone-induced PD models (Girich et al., 2020).

Trichophenol A (**150**), which originated from the endophytic fungus *Trichoderma citrinoviride* A-WH-20-3 within the marine red algae *Laurencia okamurai*, has proven to be a potent inhibitor against marine phytoplankton species such as *Chattonella marina*, *Heterosigma akashiwo*, *Karlodinium veneficum*, and *Prorocentrum donghaiense*, with IC_50_ values of 4.4, 9.1, and 5.9 μg/mL, respectively. Additionally, trichophenol A displayed broad-spectrum inhibitory effects against various marine bacteria, such as *Vibrio parahaemolyticus*, *Vibrio anguillarum*, *Vibrio harveyi*, *Vibrio splendidus*, and *Photobacterium citre*a, with MIC values ranging from 7.0 to 21 μg/mL (Liu et al., 2020). Monarubin B (**151**) and two isocoumarins (**152**-**153**), extracted from the marine bivalve-associated fungus *Monascus ruber* BB5. Compound **151** manifested robust cytotoxic effects against liver cancer cell lines HepG2 and QGY7701, with IC_50_ values of 1.72 and 0.71 μM, respectively. Compound **152** has shown moderate cytotoxicity against HepG2, QGY7701, and SUNE1 cancer cell lines, with IC_50_ values of 9.60, 7.12, and 28.12 μM. Meanwhile, compound **153** has exhibited subdued cytotoxic potential against the aforementioned cancer cell lines, with IC_50_ values recorded at 46.10, 31.62, and 39.38 μM for HepG2, QGY7701, and SUNE1, respectively (Ran et al., 2020).

The marine fungus *A. terreus* RA2905 has yielded dihydroisocoumarin (**154**), a compound that has exhibited a weak scavenging effect on DPPH radicals, with an IC_50_ value of 147 μM (Wu et al., 2020). Two isocoumarins (**155**-**156**), isolated from the marine fungus *Exserohilum* sp. CHNSCLM-0008. Compound **155** exhibited significant antimalarial potency, with an IC_50_ value of 1.13 μM, whereas compound **156** showed intermediate antimalarial efficacy at an IC_50_ value of 11.7 μM (Coronado et al., 2021). 7-hydroxyoospolactone (**157**) and parapholactone (**158**), two isocoumarin class secondary metabolites identified from the marine fungus *Paraphoma* sp. CUGBMF 180003, have both demonstrated inhibitory activity against *S. aureus*, with MIC values of 12.5 μg/mL (Xu et al., 2021). The co-cultivation of the marine fungus *Cosmospora* sp. was conducted with the plant pathogen *Magnaporthe oryzae* facilitated the extraction and isolation of two bioactive isocoumarins (**159**-**160**) with moderate inhibitory activity against *P. infestans*, *M. oryzae*, and *X. campestris*, with IC_50_ values ranging between 12.8 and 71.5 μg/mL (Oppong-Danquah et al., 2022). Derived from the marine fungus *P. citrinum* VM6, compound **161** has shown inhibitory activity against several Gram-positive bacteria, including *E. faecalis*, *S. aureus*, and *B. cereus*, in addition to the fungus *C. albicans*, with observed MIC values of 32–256 μg/mL (Anh et al., 2023).

### 4.3 Chroman

**FIGURE 8:**
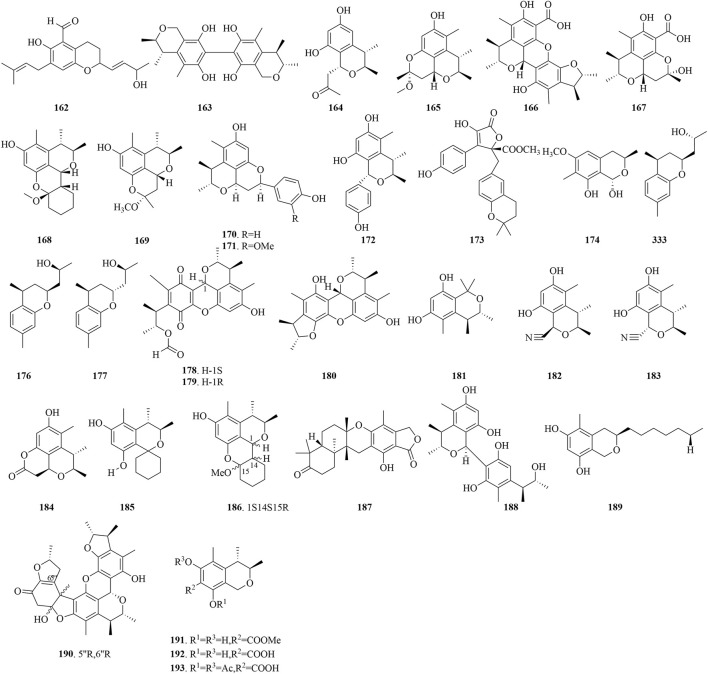
Structural formulas of the isolated compounds **162–193**.

Chroman, characterized by their benzo pyran core, constitute a class of compounds that are ubiquitous in natural products and medicinal molecules endowed with distinctive physiological properties (Ji Ram et al., 2014). The structural diversity of Chroman compounds sourced from marine fungi is elegantly presented in Figure 8, showcasing their potential as bioactive constituents with significant implications for drug discovery and development.

Chaetopyranin (**162**), from the endophytic fungus *Chaetomium globosum* of marine red algae *Polysiphonia urceolata*, has exhibited a moderate level of DPPH radical scavenging activity, with an IC_50_ value of 35 μg/mL. Moreover, it has demonstrated cytotoxicity against tumor cell lines HMEC, SMMC-7721, and A549, yielding IC_50_ values of 15.4, 28.5, and 39.1 μg/mL (Wang et al., 2006). Compound **163** discovered from the marine fungus *Aspergillus* sp. (MF-93) native to Quanzhou Bay, showed notable inhibitory efficacy against TMV at 0.2 mg/mL, achieving an inhibition rate of 35.5% (Wu et al., 2009). Penicitrinol C (**164**) and penicitrinol E (**165**), isolated from *P. citrinum*, have exhibited subtle cytotoxicity against HL-60 cells, with IC_50_ values of 52.8 and 41.2 μmol/L, respectively (Chen et al., 2011). Penicitrinol J (**166**) and penicitrinol K (**167**), isolated from *Penicillium* sp. ML226, have shown inhibition rates of 25.1% and 9.2% against the HepG-2 cell line at 10 μg/mL, respectively. Furthermore, compounds **166** and **167** have only weakly influenced *S. aureus*, yielding inhibition zone diameters of 10 mm and 9 mm at 20 μg/6 mm paper disk, respectively (Wang et al., 2013). In addition, compound **166** displayed moderate antimicrobial activity against *B. subtilis* JH642, *Bacillus megaterium* DSM32, and *Mycobacterium smegmatis* ATCC607, with corresponding MIC values of 16, 16, and 32 μg/mL. It also showed weaker inhibitory effects against *B. subtilis* DSM10 and *S. aureus* ATCC25923, with MIC values of 64 μg/mL for both strains (Sabdaningsih et al., 2020). The marine fungus *P. chrysogenum* HND11-24, sourced from mangroves, has produced compounds **165**, **168**, and **169**. Compound **168**, in particular, has exhibited potent inhibitory action against HeLa, BEL-7402, HEK-293, HCT-116, and A-549 cell lines, with IC_50_ values in the range of 4.6–10.5 μM. Furthermore, the presence of the additional cyclohexane moiety in compound **168** was suggested to enhance its activity, as compounds **165** and **169** showed comparatively weaker inhibitory activities against HeLa and HEK-293 cell lines, with IC_50_ values in the range of 45.0 to 32.6 μM and 35.5–42.5 μM, respectively (Guo et al., 2015).

Two unique tetracyclic compounds, **170** and **171**, were isolated from the co-culture of marine fungi *P. citrinum* and *B. felina*. Compound **170** exhibited inhibitory activity against *E. coli* and *S. aureus*, with MIC values of 8.0 μg/mL for both. Compound **171** displayed even lower MICs, 2.0 μg/mL for *E. coli*, and 4.0 μg/mL for *S. aureus*, suggesting that the OMe group at the C-3′ position may enhance the antimicrobial potency. Additionally, compounds **170** and **171** also showed weak activity against the aquatic pathogen *V. alginolyticus*, with a MIC value of 16.0 μg/mL for both (Meng et al., 2015). The marine fungus *P. citrinum* has yielded Penicillin L (**172**), which has demonstrated a moderate cytotoxic impact on the HL-60 cell line, with an IC_50_ value of 22.7 μg/mL. Meanwhile, compound **173**, extracted from the fungus *Eutypella* sp. found in the South China Sea gorgonian, has shown antimicrobial activity against *B. cereus* and *S. aureus*, with MICs of 6.25 and 12.5 μM (Liao et al., 2017). 3,7-dimethyl-1,8-hydroxy-6-methoxyisochroman (**174**), extracted from the marine fungus *Penicillium* sp. SF-6013, has demonstrated inhibitory effects on the LPS-induced production of prostaglandin E2 (PGE2), nitric oxide (NO), COX-2, and inducible nitric oxide synthase (iNOS) in RAW264.7 and BV2 cells, and also downregulates the mRNA expression of pro-inflammatory cytokines including IL-1β and IL-6 (Ko et al., 2019). Additionally, Trichobisabolins J-L (**175**-**177**), from the endophytic fungus *Trichoderma asperellum* A-YMD-9-2 in marine red algae *Gracilaria verrucosa*, have shown high inhibitory activity against phytoplankton species such as *C. marina*, *K. veneficum*, and *P. donghaiense*, with IC_50_ values from 0.93 to 9.2 μg/mL. The structure-activity relationship analysis implies that the phenyl group in their structure may play a role in their inhibitory activity. Additionally, compounds **184**–**186** showed weak antimicrobial activity against marine pathogens *V. anguillarum*, *V. harveyi*, *V. parahaemolyticus*, *V. splendidus*, and *Pseudoalteromonas citrea*, with inhibition zone diameters of 6.3–8.1 mm at a 40 μg paper disc for all five pathogens (Song et al., 2019).

From the deep-sea derived *P. citrinum* NLG-S01-P1, 1-epi-citrinin H1 (**178**) and its three related biogenetic compounds (**179**-**181**) have been obtained. Compound **178** has exhibited inhibitory effects against MRSA strains, similar to the control chloramphenicol, with a MIC range of 7–8 μg/mL. Meanwhile, Compounds **179**–**181** have demonstrated a weaker antimicrobial activity against *V. vulnificus*, with MIC values ranging from 32 to 50 μg/mL. Additionally, compounds **179**-**189** exhibited significant inhibitory activity against the A549 and HeLa cell lines, with IC_50_ values ranging from 17.7 to 46.3 μM (Wang et al., 2019). Cladosporins A-C (**182**-**184**) were isolated from the deep-sea derived fungus *Cladosporium* sp. SCSIO z015, which displayed LC_50_ values of 72.0, 81.7, and 49.9 μM against brine shrimp, respectively (Amin et al., 2020). Penitol A (**185**) and penicitols I (**186**), featuring a tricyclic spiro framework, have been extracted from the coral-associated fungus *P. citrinum* (Cheng et al., 2021). Compound 185 manifested cytotoxic effects against the K562 cell line, achieving an IC_50_ value of 8.8 μM. Moreover, compounds **185** and **186** displayed weak antimicrobial activity against *S. aureus*, *B. subtilis*, and vancomycin-resistant enterococci (VRE), with MIC values ranging from 16 to 32 μg/mL and 32–64 μg/mL, respectively.

The soft coral-derived *Penicillium glabrum* glmu003 has yielded australide V (**187**), a compound that has exhibited a limited inhibitory activity against pancreatic lipase (PL), as indicated by an IC_50_ value of 23.9 μg/mL (Zhang et al., 2021). Compound (**188**) was extracted from the sea star-derived fungus *Penicillium* sp. GGF 16-1-2. The compound’s efficacy against *Colletotrichum gloeosporioides* was assessed using a mycelial growth rate assay, yielding an LD_50_ of 4.34 μg/mL, which is notably lower than that of the positive control Carbendazim, with an LD_50_ of 49.58 μg/mL (Fan et al., 2022). From the co-culture of the marine fungus *Cosmospora* sp. and the plant pathogen *M. oryzae*, pseudoanguillosporins A (189) have been isolated and have demonstrated potent antimicrobial activity against *Pseudomonas syringae*, *X. campestris*, *P. infestans*, and *M. oryzae*, with IC_50_ values of 0.8–23.4 μg/mL (Oppong-Danquah et al., 2022). Dihydrocitrinin (**190**) was isolated from *P. citrinum* VM6, a fungus derived from marine sediments, which had certain antimicrobial activity and was effective against *E. faecalis*, *S. aureus*, *B. cereus* and, with MIC values ranging from 8 to 16 μg/mL (Anh et al., 2023). Neotricitrinols B (**191**), featuring a unique octacyclic framework, isolated from the fungus *P. citrinum* W23, and has been assessed for its anti-osteoporotic activity, It has demonstrated the ability to promote osteogenic mineralization in primary bone mesenchymal stem cells (BMSCs) but also to suppress adipogenic differentiation in a dose-dependent manner, indicating its robust anti-osteoporotic potential (He et al., 2023). Dihydrocitrinin (**190**), (3R,4S)-6,8-dihydroxy-3,4,5-trimethylisochromane- 7-carboxylatemethyl (**192**), and (3R,4S)-dihydrocitrinin diacetate (**193**) were isolated from the marine fungus *Penicillium* sp. TW 131-64. Compound **192** showed moderate inhibitory effects against the standard strain G27 and the clinically drug-resistant strain HP159 of *H. pylori*, with MIC values ranging from 8 to 16 μg/mL. Initial structure-activity relationship analysis indicates that the C-7 carboxylate esterification in compound **192** markedly improves its activity against *H. pylori*, conferring greater potency compared to compounds **190** and **193** (Lai et al., 2023).

### 4.4 Citrinin

**FIGURE 9:**
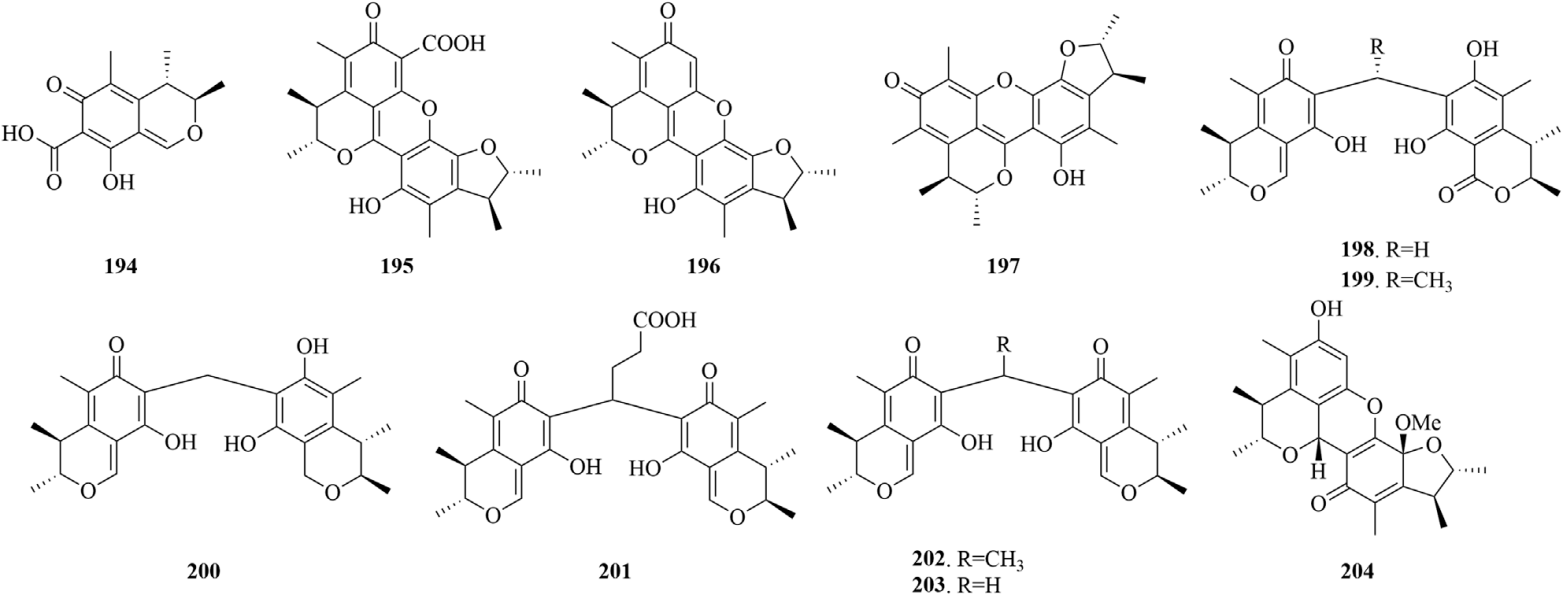
Structural formulas of the isolated compounds **194–204**.

Citrinin, recognized as a prevalent mycotoxin in the food supply, is a fungal-derived secondary metabolite with notable bioactivity (Frank, 1992). Its spectrum of biological effects is impressive, encompassing antifungal and antibacterial actions, alongside potential anticancer and neuroprotective capabilities when assessed *in vitro*. The structural diversity of citrinin analogues sourced from marine fungi is elegantly showcased in Figure 9, highlighting their potential as leads in the development of novel therapeutic agents.

Citrinin (**194**), extracted from the marine fungus *P. purpurogenum* G59, has demonstrated a notable inhibitory impact on K562 cells, achieving an IC_50_ value of 52.6 μg/mL (Chai et al., 2012). Meanwhile, Penicitrinone E (**195**), isolated from the marine *Penicillium* sp. ML226, has exhibited selective activity against HepG-2 cells at 10 μg/mL, displaying an inhibition rate of 6.3% (Wang et al., 2013). Moreover, Penicitrinone A (**196**), from the marine-derived *P. citrinum*, has manifested weak cytotoxic effects on the A549 and A375 cell lines, with IC_50_ values of 49.15 μM (Chu et al., 2021) and 65.4 μg/mL (Ni et al., 2015). Additionally, compound **196** has demonstrated antimicrobial activity, with an MIC of 32 μg/mL against *M. smegmatis* ATCC 607 (Sabdaningsih et al., 2020). Moreover, compound **196** effectively inhibits the production of superoxide anions in human neutrophils stimulated by fMLP, with an IC_50_ value of 2.67 μM (Chu et al., 2021). A citrinin dimer, penicitrinone F (**197**), extracted from the deep-sea fungus *P. chrysogenum* SCSIO 41001, has demonstrated inhibitory effects on enterovirus 71 (EV71) cells, with an IC_50_ of 14.50 μM (Chen et al., 2017).

The marine fungus *Penicillium* sp. GGF 16-1-2, found in association with sea stars, has yielded four novel carbon-bridged citrinin dimers (**198**-**201**) and two known related compounds (**202**-**203**), have been found to possess robust antibacterial activity against *C. gloeosporioides*, with LD_50_ values in the range of 0.61–16.14 μg/mL. Notably, compound **203** has shown the most potent antifungal activity, with an LC_50_ value of 0.61 μg/mL. The structure-activity relationship analysis indicated that citrinin monomers and the methylene bridge are essential for their antifungal activity, and modifications such as alkyl group introduction or an increase in the alkyl bridge length can reduce their bioactivity. Moreover, the oxidation or reduction of citrinin results in diminished antibacterial activity. Furthermore, compound **198** has demonstrated potent cytotoxic effects against human pancreatic cancer cells BXPC-3 and PANC-1, achieving IC_50_ values of 12.25 and 24.33 μM, respectively, and may induce apoptosis in BXPC-3 via the modulation of caspase-3 activation (Fan et al., 2022). Penicitriol A (**196**) and penicitriol B (**204**) were isolated from *P. citrinum* XIA-16. At a concentration of 10 μM, penicitriol A (**196**) in human lung cancer cells H1299 is capable of inhibiting copper toxicity without disrupting DLAT oligomerization, maintaining a cell viability of 68.2%. While compound 204 exerts an inhibitory effect on RSL3-induced ferroptosis in A375 melanoma cells, as evidenced by its reduction of lipid peroxidation and HO-1 levels, at an EC_50_ concentration of 2.0 μM (Yu et al., 2023).

### 4.5 Other compounds

**FIGURE 10:**
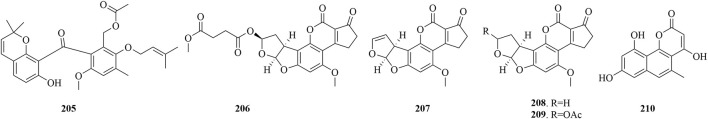
Structural formulas of the isolated compounds **205–210**.

Isolated from *A. versicolor* HDN11-84, which resides in the mangrove rhizosphere, Arugosin K (**205**) (Figure 10) has exhibited potent cytotoxic effects against the Hela, HL-60, and NB4 cell lines, achieving IC_50_ values between 9.2 and 15.2 μM (Li et al., 2017). Aflatoxin B2b (**206**) along with three known compounds (**207**-**209**) was extracted from the endophytic fungus *Aspergillus flavus* 092008, which resides within the mangrove plant *Hibiscus*. Compound **206** showed moderate antibacterial potency against *E. coli*, *B. subtilis* and *Escherichia aerogenes*, with MICs of 22.5, 1.7, and 1.1 μM, respectively. Compounds **207**-**209** also demonstrated moderate antibacterial activity against *E. aerogenes*, with MICs of 3.2, 3.2, and 2.7 μM. Notably, compound **207** exhibited weak antibacterial effects against *P. aeruginosa*, with a MIC value of 32.1 μM. The findings suggested that the esterification at the C-8 position with a longer fatty acid in aflatoxin B2b (**206**) may enhance its antibacterial activity, making it the most effective among the tested compounds. Furthermore, compound 206 has proven to be cytotoxic against the A549 and K562 cell lines, with IC_50_ values of 8.1 μM and 2.0 μM, respectively (Wang et al., 2012). Pannorin (**210**), isolated from the fungus *B. fuckeliana*, sourced from the Wadden Sea in Germany, exhibited potent inhibition of GSK-3β enzyme in an assay, with an IC_50_ value of 0.35 μM (Wiese et al., 2016).

## 5 Potential of benzopyran compounds from marine fungi in drug development

Within the spectrum of esteemed natural product frameworks, benzopyran compounds are renowned for their bioactivity. Warfarin, one of the pioneering anticoagulant drugs approved in 1954, exemplifies this with its benzo-α-pyrone (coumarin) nucleus (Pirmohamed, 2006). Similarly, methoxsalen and trioxsalen, which have garnered extensive research for their use in treating psoriasis, also incorporate a benzo-α-pyrone nucleus. The benzo-γ-pyrone (chromone) nucleus is also present in cromolyn and pranlukast, drugs that were approved for asthma treatment in 1982 and 2007, respectively. Furthermore, an array of small molecule drugs centered around the benzopyran core are currently undergoing clinical investigation, highlighting the ongoing interest and potential of these marine-derived compounds in pharmaceutical development (Sharma et al., 2024).

A growing body of research indicates that the harnessing of anticancer immune responses could be instrumental in post-treatment cancer control, potentially eradicating residual malignant cells or curbing micrometastasis. The incorporation of such immune responses into cancer therapeutic strategies, alongside the development of chemotherapeutic agents capable of both direct cytotoxicity and the induction of specific immune reactions, may substantially enhance the efficacy of cancer treatments and mitigate the risk of drug resistance. In a study by Rönsberg et al. (2013), Phomoxanthone A (**56**) demonstrated exceptional pro-apoptotic potency against a range of human cancer cell lines, with its impact on healthy blood cells being markedly reduced by an order of magnitude. This compound also stands out as a potent stimulator of mouse T lymphocytes, NK cells, and macrophages, suggesting a synergistic activation of the immune system that complements its pro-apoptotic effects. Such dual-targeted action may offer a robust approach to counteracting chemoresistance. Given the pivotal role of angiogenesis in tumor progression, angiogenesis inhibitors are emerging as novel therapeutic targets. The compound decarboxyhydroxycitrinone (**102**) (Tsukada et al., 2011), with its anti-proliferative effects on HUVECs and HUAECs and IC_50_ values of 7.6 and 17.4 μM respectively, presents itself as a promising candidate for inhibiting tumor angiogenesis, thus opening new avenues in the fight against cancer.

The advent of the SARS-CoV-2 virus at the close of 2019 marked the onset of a severe respiratory disease that has escalated into one of the most formidable pandemics of our era. COVID-19 has challenged the global community, with the efficacy of vaccines being tested by the emergence of elusive variants. The evolution of more potent genetic strains coupled with the virus’s burgeoning resistance to existing treatments has intensified the quest for potent antiviral agents against SARS-CoV-2. In a significant study by ElNaggar et al. (2022), Aurasperone A (**92**) demonstrated robust inhibitory effects against SARS-CoV-2, with an IC_50_ of 12.25 µM, rivaling the efficacy of the benchmark remdesivir at 10.11 µM. Notably, compound **92** displayed negligible cytotoxicity towards Vero E6 cells, with a CC_50_ of 32.36 µM and a selectivity index (SI) of 2,641.5, vastly outperforming remdesivir’s SI of 41.07 at a CC_50_ of 415.22 µM. Advanced screening utilizing molecular docking simulations (MDS) indicated that compound **92** possesses a high affinity for the Mpro enzyme’s active site, capable of forming numerous hydrogen bonds, water bridges, and hydrophobic interactions, culminating in stable binding over a 150 ns timeframe. These discoveries lay a solid foundation for the advancement of more efficacious Mpro inhibitors, offering new horizons in the battle against SARS-CoV-2.

Glycogen synthase kinase 3 (GSK-3) stands as a pivotal target in the quest for novel therapeutics. Involved in the intricate signaling cascades of type II diabetes, neurodegenerative conditions, oncological processes, and a spectrum of other pathological states, GSK-3’s modulation is of high interest. The inhibitory potential of compounds **134–136** against GSK-3β was evaluated in enzyme assays (Wiese et al., 2016), revealing inhibition efficacies that rivaled those of the benchmark compound TDZD-8. Alternariol (**136**) emerged as the most potent, with an IC_50_ of 0.13 μM, closely followed by compound **136** at 0.20 μM. These findings underscore the remarkable inhibitory capabilities of the trio, all characterized by a highly oxygenated benzo-coumarin core, which positions them as novel and efficacious GSK-3β inhibitors, heralding a promising avenue for the development of therapeutics targeting a myriad of diseases.

Inflammation serves as a vital immune response, instrumental in neutralizing harmful stimuli and facilitating the repair of cellular and tissue damage. Despite its protective role, unchecked acute or chronic inflammation can precipitate a host of serious conditions, including but not limited to arthritis, asthma, inflammatory bowel disease, Parkinson’s disease, Alzheimer’s disease, and sepsis. Within these pathological contexts, the regulation of inflammatory responses is paramount for therapeutic intervention. In a study by Ko et al. (2019), the anti-inflammatory mechanisms of compound **179** were elucidated, revealing its efficacy in dampening inflammation through the suppression of the nuclear factor (NF)-κB and c-Jun N-terminal kinase (JNK) MAPK signaling cascades. Furthermore, the anti-inflammatory potency of DMHM is linked to its upregulation of HO-1 via the activation of the Nrf2 pathway. By targeting multiple inflammatory pathways, compound **179** illustrates its promise as a therapeutic agent for the management of inflammatory and neurodegenerative disorders.

The differentiation of mesenchymal stem cells from bone marrow hematopoietic stem cells gives rise to both osteoblasts, pivotal in bone metabolism, and adipocytes, central to fat metabolism. In clinical scenarios of osteoporosis, there is observed an increase in adipocyte presence and a concomitant reduction in osteoblast numbers within the bone marrow. Targeting the differentiation of these stem cells to favor bone regeneration offers a strategic approach to combat osteoporosis. The osteogenic potential of neotricitrinols B (**191**) was scrutinized by He et al. (2023), revealing that compound **191** robustly promoted osteogenic mineralization in primary bone mesenchymal stem cells (BMSCs) and concurrently curbed adipogenic differentiation in a dose-responsive manner. This dual action underscores the compound’s significant anti-osteoporotic efficacy. Importantly, at concentrations below 10 μM, compound **191** exhibited negligible cytotoxic effects, highlighting its safety profile for potential therapeutic applications.

## 6 Conclusion

This comprehensive review has delineated the benzopyran compounds produced by marine fungi from the onset of the 21st century to the close of 2023, highlighting their structural diversity and biological profiles. A total of 510 benzopyran compounds were characterized, with 223 showcasing bioactivity, mainly isolated from marine fauna (44%), marine sediments (28%), and marine flora (25%). The fungal genera predominantly represented were *Penicillium* (43%) and *Aspergillus* (20%). The benzopyran compounds derived from marine fungi have attracted considerable interest due to their diverse chemical structures and pronounced bioactivity. The majority of these compounds have manifested exceptional bioactivity across a spectrum of biological functions, including antibacterial (35%), antitumor (31%), enzymatic inhibition (5%), and antiviral (3%) activities. While existing research has begun to scratch the surface of the biological potential harbored by benzopyran compounds, the vast majority of these, isolated from the marine milieu, remain an uncharted territory in terms of their full biological capabilities. The untapped reservoir of these marine-derived metabolites is ripe for exploration. It is with this in mind that we foresee a promising future for the development of these natural products, which holds the key to uncovering groundbreaking pharmaceuticals endowed with unprecedented mechanisms of action.
