# Supplementary material for: Recent advances in the structures and bioactivities of benzopyrans derived from marine fungi: a review
Source: Front Pharmacol. 2024 Oct 24;15:1482316. doi: 10.3389/fphar.2024.1482316 (PMC11540774; doi:10.3389/fphar.2024.1482316)
Supplement: Supplementary file 1 [file Table1.DOCX]

**Table S1.** Catalog of marine fungus-derived benzopyrans with significant biological activity.

| **Compound Name/ Chemical Class** | **Marine Source** | **Type of Strains** | **Activity (MIC/ Inhibition zones)** | **Reference** |
| --- | --- | --- | --- | --- |
| Aspergillitine (**1**) | Sponge-derived *Aspergillus niger* | *B. subtilis* | Inhibition zones were  7 mm at 5 µg/mL, 8 mm at 10 µg/mL | (Lin et al., 2003) 2003 |
| Compound **19**  Secalonic acid D (**20**)  Secalonic acid B (**21**)  Compound **22** | Gorgonian-derived *Penicillium* sp. SCSGAF 0023 | *B. subtilis*, *E. coli*,  *M. luteus*, *P. nigrifaciens* | 24.4-390.5 µg/mL | (Bao et al., 2013) 2013 |
| Engyodontiumones H (**35**)  Aspergillusone B (**36**)  AGI-B4 (**37**) | Sediment-derived *Engyodontium album* DFFSCS 021 | *E. coli*, *B. subtilis* | ≤64.0 µg/mL | (Yao et al., 2014) 2014 |
| JBIR-99 (**8**)  Compound **62**  Engyodontochone E-F (**63**-**64**) | Sponge-derived *Engyodontium album* Strain LF069 | *S. epidermidis*,  *S. aureus*, MRSA | 0.21-6.77 μM | (Wu et al., 2016) 2016 |
| Compound **70-71** | Sponge-derived *Streptomyces sp*. G248 | *E. faecalis*, *S.aureus*, *B.cereus*, *P. aeruginosa*, *S. enterica*, *C. albicans* | 1-16 µg/mL | (Cao et al., 2019) 2019 |
| Daidzein (**78**)  Compound **79**  Biochanin A (**80**)  Psoralenol (**81**) | Coral-derived *Aspergillus terreus* C23-3 | G+, G- | 10.0-13.8 mm at 10 µg/disk | (Yang et al., 2020a) 2020 |
| Lateropyrone (**89**) | *Taeniolella* sp. BCC31839 | *B. cereus* | 12.5 µg/mL | (Intaraudom et al., 2021) 2021 |
| Aspergilluone A (**91**) | Sponge-derived *Aspergillus* sp. LS57 | *M. tuberculosis*, S*. aureus*, *B. subtilis*, *E. coli* | 32, 64, 128, 128 μg/mL | (Liu et al., 2021) 2021 |
| Cephalochromin (**93**)  Ustilaginoidin G (**94**)  Soudanones D-E (**160**, **159**)  Pseudoanguillosporins A (**189**) | Co-cultivation of *Cosmospora* sp. and *Magnaporthe oryzae* | *P. infestans*,  *R. solanacearum*,  *X. campestris* | 0.8-71.5 µg/mL | (Oppong-Danquah et al., 2022) 2022 |
| Compound **95**  Stoloniferol B (**161**)  Dihydrocitrinin (**190**) | Sediment-derived *Penicillium citrinum* VM6 | *E. faecalis*, *S. aureus*,  *B. ceureus*, *S. enterica*,  *C. albicans* | 16-256 µg/mL | (Anh et al., 2023) 2023 |
| Compound **97** | Mangrove-derived *Trichoderma lentiforme* ML-P8-2 | *C. albican* | 25 µM | (Yin et al., 2023) 2023 |
| Aspergillumarin A-B (**104**, **103**)  Penicimarins F (**105**)  Compound **106** | Sponge-derived *Penicillium* sp. MWZ14-4 | *S. Albus, S. aureus*  *B.cereus*,  *V. parahemolyticus* | 12.5 μM  12.5 μM  6.25 μM | (Qi et al., 2013) 2013 |
| Compound **111**-**113** | Sponge-derived *Aspergillus similanensis* KUFA 0013 | AMP | Inhibition zones were 2.5-5 mm at 15 µg/disc | (Prompanya et al., 2014) 2014 |
| Asperentin (**114**) | Alga-derived *Aspergillus* sp. F00785 | *C. gleosporioides Penz*,  *C. gleosporioides* (Penz) Sacc., *B. cinerea* Pers | Inhibition zones were 19.7, 13.3, 1.67 mm at 5 mg/mL | (Tang et al., 2014) 2014 |
| Decarboxydihydrocitrinone (**125**)  Citrifelins A-B (**170**-**171**) | Mangrove-derived *Penicillium citrinum* and *Beauveria felina* | *V. alginolyticus*  *E. coli*, *S. aureus*,  *V. alginolyticus* | 8.0 µg/mL  2.0-16.0 µg/mL | (Meng et al., 2015) 2015 |
| Spiromastols I (**126**) | Sediment-derived *Spiromastix* sp. MCCC3A00308 | *X. vesicatoria*,  *P. lachrymans*, *A.tumefaciens*, *R.solanacearum*, *B.thuringensis*,  *S. aureus*, *B.subtilis* | 8-16 µg/mL | (Niu et al., 2015) 2015 |
| Pleosporalone A (**127**) | Sediment-derived *Pleosporales* sp. CF09-1 | *B. cinerea*, *R. pestici*,  *P. capsici* | 0.39, 0.78, 0.78 μM | (Cao et al., 2016) 2016 |
| Penicimarins G (**128**)  Penicimarins K (**129**) | Mangrove-derived *Penicillium citrinum* | *S. epidermidis*, *S. aureus*, *E. coli*, *B. cereus*, *V. alginolyticus* | 10-20 μM | (Huang et al., 2016a) 2016 |
| Compound **133** | Sediment-derived *Leptosphaeria* sp. SCSIO 41005 | *C. miyabeanus*, *C. albicans* | 0. 5 μM, 101 μM | (Luo et al., 2017) 2017 |
| Compound **134** | Mangrove-derived *Eurotium chevalieri* KUFA 0006 | *E. coli* | Biofilm biomass were 57.6% at 64 µg/mL | (May Zin et al., 2017) 2017 |
| Compound **136**  Compound **138**-**139** | Mangrove-derived *Ascomycota* sp. CYSK-4 | *S. aureus*, *E. coli*,  *K. pneumoniae*  *S. aureus*, *B. subtilis*,  *E. coli*, *K. pneumoniae*,  *A. calcoaceticus* | 50 µg/ mL  25-50 µg/mL | (Chen et al., 2018) 2018 |
| Penicitol D (**144**)  (3S,4S)-sclerotinin A (**145**)  Compound **178**-**181** | *Penicillium citrinum* NLG-S01-P1 | MRSA  *V. vulnificus*,  *V. campbellii*, G27, HP159, MRSA,  *V. vulnificus* | 7-8 µg/mL  15-17 µg/mL  7-50 μg/mL | (Wang et al., 2019) 2018 |
| Botryospyrones A-C (**146-148**) | Mangrove-derived *Botryosphaeria ramosa* L29 | *F. oxysporum*, *P. italicum*,  *F. graminearum* | 105.8-450.4 µM | (Wu et al., 2019) 2019 |
| Trichophenol A (**150**) | Alga-derived *Trichoderma citrinoviride* A-WH-20-3 | *C. marina*, *H. akashiwo*,  *P. donghaiense*,  *V. parahaemolyticus,*  *V. anguillarum*,  *V. harveyi*, *V. splendidus*, *P. citrea* | 4.4-21 µg/mL | (Liu et al., 2020) 2020 |
| Compound **157**  Parapholactone (**158**) | Marine mud-derived *Paraphoma* sp. CUGBMF 180003 | *S. aureus* | 12.5 µg/mL | (Xu et al., 2021) 2021 |
| Penicitrinol J (**166**)  Penicitrinol K (**167**) | Sediment-derived *Penicillium* sp. ML226 | *B. subtilis*, *B. megaterium*, *M. smegmatis*, *B. subtilis*, *S. aureus* | 16-64 μg/mL  Inhibition zones were 9 mm at 20 μg/6 mm paper disk | (Wang et al., 2013) 2013 |
| Butyrolactones V (**173**) | Gorgonian-derived *Eutypella* sp. | *B. cereus*, *S. aureus* | 6.25,12.5 μM | (Liao et al., 2017) 2017 |
| Trichobisabolins J-L(**175**-**177**) | Alga-derived *Trichoderma asperellum* A-YMD-9-2 | *C. marina*, *K. veneficum*, *P. donghaiense*  *V. anguillarum*, *V. harveyi*, *V. parahaemolyticus*,  *V. splendidus*, *P. citrea* | 0.93-9.2 μg/Ml  Inhibition zones were 6.5-8.1 mm at 40 μg/disk | (Song et al., 2019) 2019 |
| Penitol A (**185**)  Penicitols I (**186**) | Coral-derived *Penicillium citrinum* | *S. aureus*, *B. subtilis*, VRE | 16-64 μg/mL | (Cheng et al., 2021) 2021 |
| Penidicitrinin B (**188**)  Dicitrinones G-J (**198**-**201**)  Dicitrinones I-J (**202**-**203**) | Starfish-derived *Penicillium* sp. GGF 16-1-2 | *C. gloeosporioides* | 0.61-16.14 µg/mL | (Oppong-Danquah et al., 2022) 2022 |
| Compound **192**  Compound **193** | Sediment-derived *Penicillium* sp. TW 131-64 | G27, HP159 | 8-16 μg/mL  Not reported | (Lai et al., 2023) 2023 |
| Penicitrinone A (**196**) | Sponge-derived *Penicillium citrinum* WK-P9 | *M. smegmatis* | 32 μg/mL | (Sabdaningsih et al., 2020) 2020 |
| Compound **206**-**209** | Mangrove-derived *Aspergillus flavus* 092008 | *E. coli*, *B. subtilis*,  *E. aerogenes* | 1.1-22.5 µM | (Wang et al., 2012) 2012 |
| **Compound Name/ Chemical Class** | **Marine Source** | **Cell lines** | **Activity(IC_50_/EC_50_/ ED_50_/inhibition rate)** | **Reference** |
| Monodictyochromes A (**3**)  Monodictyochromes B (**4**) | Algicolous-derived *Monodictys putredinis* | CYP1A, NAD(P)H, QR | 5.3-24.8 μM | (Pontius et al., 2008) 2008 |
| JBIR-97 (**6**)  JBIR-98 (**7**)  JBIR-99 (**8**) | Sponge-derived *Tritirachium* sp. | HeLa, ACC-MESO-1 | 11.31 μM  17.63 μM  17.59 μM | (Ueda et al., 2010) 2010 |
| Janthinone (**9**)  Citrinin (**194**) | *Penicillium purpurogenum* G59 | K562 | Inhibitory rate 34.6% at 100 µg/mL  52.6 µg /mL | (Chai et al., 2012) 2012 |
| Compound **10**  Compound **11** | Seastar-derived *Trichoderma* sp. | MCF-7 | 7.82 μg/mL  9.51 μg/mL | (Lan et al., 2012) 2012 |
| Oxalicumones A-C (**12**-**14**)  Compound **15**-**18** | Gorgonian-derived *Penicilliu macum* SCSGAF 0023 | A375, A549, HeLa, HepG2, SW-620, L-02 | 11.7-281.5 μM | (Sun et al., 2012) 2012 |
| Oxalicumones A-B (**12**-**13**)  Oxalicumones D (**25**)  Oxalicumones E (**26**)  Compound **27**-**34** | Gorgonian-derived *Penicillium* sp. SCSGAF 0023. | H1975, U937, K562, BGC823, MOLT-4, MCF-7, HL60, Huh-7 | <10 μM | (Bao et al., 2014) 2014 |
| Engyodontiumones H (**35**)  AGI-B4 (**37**) | Sediment-derived *Engyodontium album* DFFSCS 021 | U937 | <10 μM | (Yao et al., 2014) 2014 |
| Penixanacid A (**38**)  Penicitols B-C (**115**-**116**)  Penicitols A (**168**)  Penicitrinols D-E (**169**,**165**) | Mangrove-derived *Penicillium chrysogenum* HND11-24 | HeLa, BEL-7402, HEK-293, HCT-116, A549 | 30.4-40.5 μM | (Guo et al., 2015) 2015 |
| Versixanthones A-C (**39**-**41**)  Compound **20** and **42-44** | Mangrove-derived *Aspergillus versicolor* HDN1009 | HL-60, K562, HL-60, K562, A549, H197, 803, HO-8910, HCT-116 | 2.6-18.2 μM  0.7-14.0 μM | (Wu et al., 2015) 2015 |
| Compound **45**-**50** | *Penicillium purpurogenum* G59 | K562, HL-60, HeLa, BGC-823 | Inhibitory rate 11.4-80.0% at 100 µg/ml | (Xia et al., 2015) 2015 |
| Penimethavone A (**51**) | Gorgonian-derived *Penicillium chrysogenum* | HeLa, RD | 8.41,8.18 µM | (Hou et al., 2016) 2016 |
| JBIR-99 (**8**)  Engyodontochone B (**62**) | Sponge-derived *Engyodontium album* Strain LF069 | NIH3T3 | 13 µM | (Wu et al., 2016) 2016 |
| Versicones G (**65**)  Arugosin K (**205**) | Mangrove-derived *Aspergillus versicolor* HDN11-84 | Hela, HL-60, NB4 | 9.2-21.7 µM | (Li et al., 2017) 2017 |
| Compound **70**, **72** | Sponge-derived *Streptomyces* sp. G248 | KB, Hep G2, Lu-1, MCF-7 | 2.0-14.6 µg/ml | (Cao et al., 2019) 2019 |
| Penixanthones C-D (**74**-**75**) | Mangrove-derived *Penicillium* sp. SCSIO041218 | K562, MCF-7, Huh-7 | 55.2-67.5 µM | (Huang et al., 2019) 2019 |
| Epiremisporine B (**46**)  Epiremisporine E (**83**) | *Penicillium citrinum* BCRC 09F0458 | A549 | 32.29 µM  43.82 µM | (Chu et al., 2021) 2021 |
| Compound **96** | Mangrove-derived *Trichoderma lentiforme* ML-P8-2 | A549 | 47.2 µM | (Yin et al., 2023) 2023 |
| 5-carboxymellein (**98**) | *Halorosellinia oceanica* BCC 5149 | KB, BC-1 | 3 μg/mL | (Chinworrungsee et al., 2001) 2001 |
| AI-77-B (**99**)  AI-77-F (**100**)  Sg17-1-4 (**101**) | Alga-derived *Alternaria tenuis* Sg17-1 | A375-S2, Hela | 0.02-0.4 mM | (Huang et al., 2006) 2006 |
| Citrinolactone D (**107**)  Penicitrinol J-K (**166**-**167**)  Penicitrinone E (**195**) | Sediment-derived *Penicillium* sp. ML226 | HeLa, HepG-2  HeLa  HepG-2 | Inhibitory rate 4% and 16.1% at 10 µg/mL  Inhibitory rate 25.1% and 9.2% at 10 µg/mL  Inhibitory rate 6.3% at 10 µg/mL | (Wang et al., 2013) 2013 |
| Bipenicilisorin (**132**) | Sediment-derived *Penicillium chrysogenum* SCSIO 41001 | K562, A549, Huh-7 | 2.59-6.94 μM | (Chen et al., 2017) 2017 |
| Penicitol D (**144**)  Compound **178**-**181** | *Penicillium citrinum* NLG-S01-P1 | HeLa  A549, HeLa | 4.1 μM  17.7-46.3 μM | (Wang et al., 2019) 2019 |
| Monarubins B (**151**)  Lunatinin (**152**)  Compound **153** | Shellfish-derived *Monascus ruber* BB5 | HepG2, QGY7701, SUNE1 | 0.71-46.10 µM | (Ran et al., 2020) 2020 |
| Chaetopyranin (**162**) | Alga-derived *Polysiphonia urceolata* | HMEC, SMMC-7721, A549 | 5.4-39.1 µg/mL | (Wang et al., 2006) 2006 |
| Penicitrinols C (**164**)  Penicitrinols E (**165**) | Sediment-derived *Penicillium citrinum* | HL-60 | 52.8 µmol/L  41.2 µmol/L | (Chen et al., 2011) 2011 |
| Penicitrinol L (**172**)  Penicitrinone A (**196**) | Sediment-derived *Penicillium citrinum* | HL-60  A375 | 22.7 μg/mL  65.4 μg/mL | (Ni et al., 2015) 2015 |
| Penitol A (**185**) | Coral-derived *Penicillium citrinum* | K562 | 8.8 μM | (Cheng et al., 2021) 2021 |
| Dicitrinones G (**198**) | Starfish-derived *Penicillium* sp. GGF 16-1-2 | BXPC-3, PANC-1 | 12.25, 24.33 μM | (Fan et al., 2022) 2022 |
| Aflatoxin B_2b_ (**206**)  Compound **206**-**207**, **209** | Mangrove-derived *Aspergillus flavus* 092008 | A549, K562  L02 | 1.1-22.5 µM  4.2-6.4 µM | (Wang et al., 2012) 2012 |
| **Compound Name/ Chemical Class** | **Marine Source** | **Target Enzyme** | **Activity (IC_50_/ inhibitory rate)** | **Reference** |
| Phomopsichins A-D (**52**-**55**)  Phomoxanthone A (**56**) | Mangrove-derived *Phomopsis* sp. 33# | AchE, α-glucosidase | Inhibitory rate 2.7-38.4% at 250 μmol | (Huang et al., 2016b) 2016 |
| Aspergivones B (**67**) | Gorgonian-derived *Aspergillus candidus* | α-glucosidase | 244 μg/mL | (Ma et al., 2017) 2017 |
| Genistein (**77**) | Coral-derived *Aspergillus terreus* C23-3 | AchE | 42.5±3.1 µM | (Yang et al., 2020a) 2020 |
| Penithochromones I-T (**84**-**86**)  Compound **87**-**88** | Sediment-derived *Penicillium thomii* YPGA3 | α-glucosidase | 268-1017 µM | (Han et al., 2021) 2021 |
| Lateropyrone (**89**) | *Taeniolella* sp. BCC31839 | NCI-H187 | 23.66 µg/mL | (Intaraudom et al., 2021) 2021 |
| Compound **96**  Compound **97** | Mangrove-derived *Trichoderma lentiforme* ML-P8-2 | AchE | 38.6 µM  20.6 µM | (Yin et al., 2023) 2023 |
| Compound **117** | Mangrove-derived *Botryosphaeria* sp. KcF6 | COX-2 | 6.51 µM | (Ju et al., 2015) 2015 |
| Compound **124** | Mangrove-derived*Aspergillus* sp. 16-5B | α-glucosidase | 90.4 μM | (Liu et al., 2015) 2015 |
| Alternariol (**130**)  Alternariol-9-methylether (**131**) | *Aspergillus* sp. LF660 and *Botryotinia fuckeliana* KF666 | GSK-3β | 0.13 μΜ  0.20 μM | (Wiese et al., 2016) 2016 |
| AustalideV (**187**) | Coral-derived *Penicillium glabrum* glmu003 | PL | 23.9 μg/mL | (Zhang et al., 2021) 2021 |
| **Compound Name/ Chemical Class** | **Marine Source** | **Target** | **Activity (IC_50_/**  **inhibitory rate)** | **Reference** |
| TMC-256C1 (**57**) | Sponge-derived *Aspergillus* sp. SF6354 | Upregulation of HO-1 and Nrf2 | Not reported | (Kim et al., 2016) 2016 |
| Psoralenone (**76**) | Coral-derived *Aspergillus terreus* C23-3 | Anti-inflammatory | Inhibitory rate 36% at 32 µM | (Yang et al., 2020a) 2020 |
| Epiremisporine B (**46**)  Epiremisporine D (**82**)  Epiremisporine E (**83**) | Marine-derived *Penicillium citrinum* BCRC 09F0458 | Decrease the production of superoxide anions | 6.39 µM  8.28 µM  3.62 µM | (Chu et al., 2021) 2021 |
| Compound **118-123** | Alga-derived *Aspergillus* sp. SF-5974 and *Aspergillus* sp. SF-5976 | Inhibit the production of NO and PGE2 | 20-61 µM | (Kim et al., 2015) 2015 |
| Compound **136**-**139** | Mangrove-derived *Ascomycota* sp. CYSK-4 | Inhibit the production of NO | 15.8-62.7 µM | (Chen et al., 2018) 2018 |
| Ochratoxin A1 (**140**)  Ochratoxin B (**141**)  Ochratoxin B methyl ester (**142**) | Sponge-derived *Aspergillus ochraceopetaliformis* | Inhibit the expression of IL-6, TNF-α | Inhibitory rates of 67.7% to 91.6% at 10 μM | (Liu et al., 2018) 2018 |
| Compound **174** | *Penicillium* sp. SF-6013 | Inhibit the production of PGE2, NO, COX-2, iNOS | Not reported | (Ko et al., 2019) 2019 |
| **Compound Name/ Chemical Class** | **Marine Source** | **Target** | **Activity (IC_50_/ inhibitory rate)** | **Reference** |
| TMC-256A1 (**2**) | *Aspergillus niger* | Against IRES and Cap-Dependent Translation | 44 μM and 80 μM | (Ovenden et al., 2004) 2004 |
| Asperxanthone (**5**)  Asperbiphenyl (**163**) | Seawater-derived *Aspergillus* sp. MF-93 | TMV | inhibitory rates 62.9% and 35.5% at 0.2 mg/mL | (Wu et al., 2009) 2009 |
| Compound **19** | Gorgonian-derived *Penicillium* sp. SCSGAF 0023 | *B.amphitrite* larvae | 6.71 mg/mL | (Bao et al., 2013) 2013 |
| Anhydrofulvic acid (**23**)  Citromycetin (**24**) | Sponge-derived *Penicillium* sp. JF-55 | PTP1B | 1.90 μM  Not reported | (Lee et al., 2013) 2013 |
| Aspergillusone B (**36**) | Sediment-derived *Engyodontium album* DFFSCS 021 | Barnacle *B. amphitrite* | 19.1 μg/mL | (Yao et al., 2014) 2014 |
| Phomopsichins A-D (**52**-**55**)  Phomoxanthone A (**56**) | Mangrove-derived *Phomopsis* sp. 33# | DPPH, OH | Inhibitory rate 3.5-40.0% at 1 mM | (Huang et al., 2016b) 2016 |
| TMC-256A1 (**2**)  Bromoflavasperone (**58**)  Flavasperone (**59**)  Fonsecin (**60**)  aurasperone B (**61**) | Mudflat-derived *Aspergillus niger* | DPPH | 0.01-25 μM | (Leutou et al., 2016) 2016 |
| Epiremisporine B (**46**)  Coniochaetone J (**66**) | Sediment-derived *Penicillium* sp. SCSIO Ind16F01 | EV71, H3N2  EV71 | 19.8 µM, 24.1 µM  81.6 µM | (Liu et al., 2017) 2017 |
| Arthones C (**68**)  Compound **69** | Sediment-derived *Arthrinium* sp. UJNMF0008 | DPPH, ABTS | 16.9 µM, 18.7 µM  22.1 µM, 18.0 µM | (Bao et al., 2018) 2018 |
| Compound **72-73** | Sponge-derived *Streptomyces sp*. G248 | *M. tuberculosis* H37Rv | 6.0, 11.1 µg/mL | (Cao et al., 2019) 2019 |
| Genistein (**77**)  Biochanin A (**80**)  Psoralenol (**81**) | Coral-derived *Aspergillus terreus* C23-3 | Larvicidal | 11.6-68.2 µM | (Yang et al., 2020a) 2020 |
| Lateropyrone (**89**) | *Taeniolella* sp. BCC31839 | Antiplasmodial | 9.75 µg/mL | (Intaraudom et al., 2021)2021 |
| Pestalotiochromones A (**90**) | Alga-derived *Pestalotiopsis neglecta* SCSIO41403 | LXR agonist | 6.2 µM | (Liang et al., 2021) 2021 |
| Aurasperone A (**92**) | *Aspergillus niger* | SARS-CoV-2  Vero E6 | 12.25 µM  32.36 µM | (ElNaggar et al., 2022) 2022 |
| 5-carboxymellein (**98**) | Marine-derived *Halorosellinia oceanica* BCC 5149 | Antimalarial | 4 µg/mL | (Chinworrungsee et al., 2001) 2001 |
| Compound **102** | Sponge-derived *Arthrinium sacchari* | HUVECs, HUAECs | 7.6, 17.4 μM | (Tsukada et al., 2011) 2011 |
| Compound **108**-**110** | *Aspergillus* sp. SCSGAF0093 | Brine shrimp lethality | 4.14, 13.74, 2.59 μM | (Xu et al., 2013) 2013 |
| Asperentin B (**135**) | Sediment-derived *Aspergillus sydowii* | PTP1B | 2.05 μM | (Wiese et al., 2017) 2017 |
| 7-O-demethylmonocerin (**143**) | Sponge-derived *Setosphaeria* sp. SCSIO 41009 | DPPH | 38 μM | (Pang et al., 2018b) 2018 |
| Demethylcitreoviranol (**149**) | Alga-derived *Penicillium* sp. KMM 4672 | Mitigated the ROS increase | Not reported | (Girich et al., 2020) 2020 |
| Compound **154** | Sea hare-derived *Aspergillus terreus* RA 2905 | DPPH | 147 μM | (Wu et al., 2020) 2020 |
| Monocerin (**155**)  Compound **156** | *Exserohilum* sp. CHNSCLM-0008 | Antiplasmodial | 1.13 μM  11.7 μM | (Coronado et al., 2021) 2021 |
| Chaetopyranin (**162**) | Alga-endophytic *Polysiphonia urceolata* | DPPH | 35 μg/mL | (Wang et al., 2006) 2006 |
| Cladosporins A-C (**182**-**184**) | Sediment-derived *Cladosporium* sp. SCSIO z015 | Brine shrimp lethality | 49.9-81.7 μM | (Amin et al., 2020) 2020 |
| Neotricitrinols B (**191**) | *Penicillium citrinum* W23 | Anti­osteoporosis | Enhanced osteogenic mineralization and suppress adipogenic differentiation | (He et al., 2023) 2023 |
| Penicitrinone F (**197**) | Sediment-derived *Penicillium chrysogenum* SCSIO 41001 | EV71 | 14.50 μM | (Chen et al., 2017) 2017 |
| Penicitrinol B (**204**) | Deep-sea gammarid shrimp-derived *Penicillium citrinum* XIA-16 | Inhibited RSL3-induced ferroptosis | 2.0 μM | (Yu et al., 2023) 2023 |
